# Identification of knowledge gaps in whole-genome sequence analysis of multi-resistant thermotolerant *Campylobacter* spp.

**DOI:** 10.1186/s12864-024-10014-w

**Published:** 2024-02-08

**Authors:** Michael Zarske, Huong Quynh Luu, Carlus Deneke, Marie-Theres Knüver, Maja Thieck, Ha Thi Thu Hoang, Nancy Bretschneider, Ngoc Thi Pham, Ingrid Huber, Kerstin Stingl

**Affiliations:** 1 Department of Biological Safety, Federal Institute for Risk Assessment (BfR), Diedersdorfer Weg 1, Berlin, D-12277 Germany; 2 National Institute of Veterinary Research (NIVR), 86 Truong Chinh Street, Hanoi, Dong Da District Vietnam; 3 Department of Bacteriology, National Institute of Hygiene and Epidemiology (NIHE), 1 Yersin Street, Hanoi, Trung District Vietnam; 4 Department of Molecular Biology and Gene Technology, Bavarian Health and Food Safety Authority, Oberschleissheim, D-85764 Germany

**Keywords:** NGS, Susceptibility testing, Antibiotic resistance, Long-read sequencing, Multidrug resistance islands, AMR, Mosaic genes, Resistance monitoring, Southeast Asia

## Abstract

**Background:**

*Campylobacter* spp. is the most frequent cause of bacterial food-borne gastroenteritis and a high priority antibiotic resistant bacterium according to the World Health Organization (WHO). European monitoring of thermotolerant *Campylobacter* spp. does not reflect the global burden of resistances already circulating within the bacterial population worldwide.

**Methods:**

We systematically compared whole genome sequencing with comprehensive phenotypic antimicrobial susceptibility, analyzing 494 thermotolerant *Campylobacter* poultry isolates from Vietnam and Germany. Any discrepancy was checked by repeating the wet lab and improving the dry lab part. Selected isolates were additionally analyzed via long-read Oxford Nanopore technology, leading to closed chromosomes and plasmids.

**Results:**

Overall, 22 different resistance genes and gene variants (e. g. *erm*(B), *aph(3’)-IIIa*, *aph(2’’)-If*, *catA*, *lnu*(C), *bla*_OXA_, *sat4*) and point mutations in three distinct genes (*gyrA*, 23S rRNA, *rpsL*) associated with AMR were present in the *Campylobacter* isolates. Two AMR genes were missing in the database and one falsely associated with resistance. Bioinformatic analysis based on short-read data partly failed to identify *tet*(O) and *aadE*, when the genes were present as duplicate or homologous gene variants. Intriguingly, isolates also contained different determinants, redundantly conferring resistance to chloramphenicol, gentamicin, kanamycin, lincomycin and streptomycin. We found a novel *tet*(W) in tetracycline sensitive strains, harboring point mutations. Furthermore, analysis based on assemblies from short-read data was impaired to identify full length phase variable *aad9*, due to variations of the poly-C tract within the gene. The genetic determinant responsible for gentamicin resistance of one isolate from Germany could not be identified. GyrT86I, presenting the main determinant for (fluoro-)quinolone resistance led to a rare atypical phenotype of ciprofloxacin resistance but nalidixic acid sensitivity. Long-read sequencing predicted AMR genes were mainly located on the chromosome, and rarely on plasmids. Predictions from long- and short-read sequencing, respectively, often differed. AMR genes were often organized in multidrug resistance islands (MDRI) and partially located in proximity to transposase genes, suggesting main mobilization of resistance determinants is via natural transformation and transposition in *Campylobacter*.

**Conclusions:**

The results of this study suggest that there is frequent resistance gene duplication, mosaicism, and mutation leading to gene variation and truncation in *Campylobacter* strains that have not been reported in previous studies and are missing from databases. Furthermore, there is a need for deciphering yet unknown resistance mechanisms and resistance spread in thermotolerant *Campylobacter* spp. that may pose a challenge to global food safety.

**Supplementary Information:**

The online version contains supplementary material available at 10.1186/s12864-024-10014-w.

## Background

Spread of multidrug-resistant bacteria is a global public health threat, contributing to more than 670,000 diseases and 33,000 deaths annually in the European Union/European Economic Area (EU/EEA) [1]. Thermotolerant *Campylobacter* species are not yet under strict control through the implementation of a safety criterion but constitute the most common bacterial cause of gastroenteritis in the European Union (EU), with around 220,000 official cases in 2019 [2]. A study estimated the true incidence of campylobacteriosis to be 47 times (95% CI 14–117) higher than reported in the EU but varying considerably between member states [3]. In the EU in 2020, a slightly lower number of campylobacteriosis cases (21%) were hospitalized compared to *Salmonella* infections (29.9%) [4]. Epidemiological data on campylobacteriosis in Vietnam is scarce due to lack of surveillance programs. *Campylobacter* spp. accounted for the largest proportion of all isolates in Vietnamese rural children with diarrheal disease [5]. Furthermore, 20% of stool samples from infants with acute diarrhea in southern Vietnam were tested positive for *Campylobacter* spp. [6].

Acute campylobacteriosis is characterized by watery and bloody diarrhea, abdominal cramps, fever and nausea [7]. In addition, long-term autoimmune sequelae might occur such as the Guillain-Barré syndrome in 0.07%, reactive arthritis in approximately 1–5% and irritable bowel syndrome in around 4% of acute cases [8]. These long-term diseases caused by campylobacteriosis contribute to a public health burden largely underestimated by the public.

A recent study showed that 31% of the reported campylobacteriosis cases were treated with antibiotics, mainly ciprofloxacin and macrolides [9]. According to the World Health Organization (WHO), *Campylobacter* spp. are high-priority antibiotic-resistant pathogens, particularly with regard to their fluoroquinolone resistance [10].

*C. jejuni* and *C. coli* asymptomatically colonize the intestinal tract of various animal species, both wild and domestic, which constitutes a potential reservoir for human infections. In particular, poultry is recognized as major source of *Campylobacter* spp. infections in humans, most probably via the consumption of cross-contaminated food during handling of raw meat or direct animal contact [11]. Zoonosis monitoring in Germany revealed a high prevalence of 51.8% *Campylobacter* spp. positive fresh chicken meat in 2020 [12]. Likewise, 31% of the tested chicken meat from Hanoi was contaminated with thermotolerant *Campylobacter* spp. [13]. Previous studies showed that *Campylobacter* isolates from Vietnam were frequently resistant to (fluoro-)quinolones (62.5–95%) and tetracyclines (71.4–75%), moderately frequent to frequently resistant to streptomycin (21.4–62.5%), and rarely to less frequently resistant to erythromycin (7.4–25%) and gentamicin (7.1–25%) [14–16]. In Germany, recent results from the 2020 zoonosis monitoring program from broiler ceca [12] revealed frequent resistance of *C*. spp. to ciprofloxacin (83.4% for *C. jejuni* and 81% for *C. coli*) and tetracycline (66.4% for *C. jejuni* and 69% for *C. coli*). All broiler isolates from cecal content were sensitive to gentamicin. Resistances to macrolides were only observed in *C. coli* isolates (17.2%). Streptomycin resistance was higher in *C. jejuni* (35%) than in *C. coli* (3.4%), which was a new observation compared to the previous years [17, 18].

Increasing occurrence of antimicrobial resistance (AMR), impeding the effectiveness of antibiotics used for treatment of bacterial diseases, poses a threat to global health [19]. Use of antimicrobials in animal production is recognized as one of the drivers of AMR [20, 21]. In order to reduce the spread of antibiotic resistance in animal production, livestock farms in Germany have been obliged to report and reduce their use of antibiotics since 2011. The overall significant decrease of antibiotic use in all farm animals by 31.6% between mid-2014 compared to mid-2017 was only marginally reflected in the poultry production chain, with a maximum reduction of 3.8% observed in turkey production [22]. From 2017 until 2021, antibiotic use in poultry was significantly reduced by 11.5% in chicken and 13.1% in turkey, while during the same time period antibiotic use in all animals was reduced by 18.2% [23]. In Vietnam, antimicrobial use in livestock accounted for 71.7% (2,751 t) of the total antimicrobials used in 2015. This corresponded to nearly the same amount of antimicrobials per kg of biomass used for human and animal treatment and a 1.6-fold higher use compared to the EU [24]. Some of the antimicrobials used in both countries were among the “highest priority critically important antimicrobials” defined by WHO, i.e. (fluoro-)quinolones, polypeptide antibiotics and macrolides [25–28].

Systematic analysis and reliable diagnostics of multi-resistant bacterial pathogens are essential to prevent their global spread. A number of studies, delivering whole genome sequencing data with some phenotypic analysis of thermotolerant *Campylobacter* spp. have previously been published [29–35]. However, rigorous in-depth analyses, aiming to identify and solve discrepancies between whole genome sequencing data and phenotypic resistance profiles are scarce for *Campylobacter* spp. Here, we evaluated a common strategy, the prediction of AMR resistant determinants by AMRFinderPlus based on short-read assembly data by recording concordances and experimentally re-analyzing discrepancies between pheno- and genotype of nearly 500 thermotolerant *Campylobacter* spp. from Germany and Vietnam. A selection of isolates was also processed by long-read sequencing using the Oxford Nanopore Technology. The study aimed at identifying knowledge gaps to be addressed in order to use WGS as a tool to reliably predict AMR in *Campylobacter* spp. In particular, it should be clarified, which specific features of AMR in *Campylobacter* spp. still pose problems for current routine WGS analysis and have to be addressed in the future.

## Methods

### Isolates and growth conditions

*C. coli* and *C. jejuni* isolates from Germany were isolated within the zoonosis monitoring program from different poultry matrices from 2013 to 2021 by the federal state laboratories according to EN ISO 10272-1 valid in the respective year [36, 37]. Isolates from Vietnam were isolated from fresh chicken feces from primary production and chicken meat from retail in Hanoi and Haiphong between 11/2016 and 03/2018 by the National Institute of Veterinary Research (Hanoi, Vietnam) by direct streaking on modified charcoal cefoperazone deoxycholate agar (mCCDA, Thermo Fisher Scientific Inc., Waltham, MA, USA) according to EN ISO 10272-1:2017 [37]. At the National Reference Laboratory, isolates were subcultured on Columbia agar supplemented with 5% sheep blood (Oxoid, Thermo Fisher Scientific Inc., Waltham, MA, USA) (ColbA) or passaged in Bolton broth (Oxoid, Thermo Fisher Scientific Inc.) and subcultured on mCCDA in case isolates still exhibited non-*Campylobacter* background flora. Incubation was performed for 48 h under microaerobic conditions (5% O_2_, 10% CO_2_, rest N_2_) at 42 °C. The isolates were stored at − 80 °C using the cryobank system (Mast Diagnostica GmbH, Reinfeld, Germany). For DNA extraction and antibiotic susceptibility testing isolates from − 80 °C stock cultures were grown on ColbA for 24 h under microaerobic conditions at 42 °C and subcultured once for another 20 ± 2 h prior to use.

### Species differentiation by PCR

DNA of the isolates was extracted by resuspension of a quarter 10 µL loop of cell material in 400 µL Tris-EDTA buffer (1 mM Tris, 0.1 mM sodium ethylenediaminetetraacetic acid at pH 8.0) followed by 1:100 dilution in 5% Chelex 100 resin (Bio-Rad Laboratories GmbH, Feldkirchen, Germany). Subsequently, thermal lysis was performed for 15 min at 95 °C. After centrifugation at 14,000 x g at 4 °C for 10 min, 2.5 µl of the supernatant was used for real-time PCR analysis, targeting specific fragments of the *C. jejuni mapA*, the *C. coli ceuE* and the *C. lari glyA* genes [38, 39].

### Antibiotic susceptibility testing by microdilution

Broth microdilution susceptibility testing was performed according to M45-A and VET06 [40, 41]. Strains subcultured for 24 ± 2 h at 42°C on ColbA were inoculated in cation-supplemented Mueller-Hinton broth (Thermo Fisher Scientific Inc., Waltham, MA, USA) with 5% fetal calf serum (PAN-Biotech, Aidenbach, Germany) (CAMHB/FCS) at a bacterial concentration of 2–8 × 10^5^ CFU/ml. For this purpose, bacteria were suspended at an OD_600_ of 0.2 in buffered peptone water (10 g/L peptone, 5 g/L NaCl, 9 g/L Na_2_HPO_4_ × 12 H_2_O, 1.5 g/L KH_2_PO_4_, pH 7.0 ± 0.2 at 25°C), which corresponds to approximately 5 × 10^8^ CFU/ml [42]. Upon a 10^− 3^ dilution in CAMHB/FCS, 100 µl of the resulting 5 × 10^5^ CFU/ml were used as inoculum per well. The inoculum was occasionally controlled by plating 100 µl of a further 10^− 3^ dilution in duplicate on ColbA in order to obtain approximately 50 CFU per plate. Minimum inhibitory concentrations were determined using the European standardized EUCAMP2 plate (Thermo Fisher Scientific Inc., Waltham, MA, USA). In addition, custom plate formats were prepared with the following antimicrobial agents (Sigma Aldrich, St. Louis, MO, USA) and their concentration ranges: ampicillin (0.5–512 mg/L), chloramphenicol (2-128 mg/L), florfenicol (0.25-16 mg/L), kanamycin (2-1024 mg/L), lincomycin (0.25–128 mg/L), nourseothricin (mixture of streptothricins C, D, E and F; 1-512 mg/L) and spectinomycin (2-512 mg/L). Stock solutions of the antimicrobials were prepared in H_2_O, for florfenicol in dimethyl sulfoxide, and for chloramphenicol in ethanol. The microtiter plates with U-bottom (Greiner Bio-One International GmbH, Frickenhausen, Germany) were prepared one day in advance by adding 50 µl CAMHB/FCS supplemented with the respective double-concentrated antimicrobial per well and stored sealed at 5°C before inoculation. Test strains were prepared as described above except that the inoculum was double-concentrated in a volume of 50 µL (1 × 10^6^ CFU/ml), which was added to each well of the custom plates, already loaded with 50 µl of double-concentrated antimicrobial per well. Samples were incubated at 37°C for 44 ± 4 h under microaerobic conditions. Minimal inhibitory concentrations (MICs; in mg/L) were semi-automatically analyzed using the Sensititre™ Vizion™ system (Thermo Fisher Scientific Inc., Waltham, MA, USA) and the Sensivizion V2.0 software (MCS Diagnostics BV, Swalmen, The Netherlands). Epidemiological cut-off values (ECOFFs, Table 1) for resistance determination were based on the European Committee on Antimicrobial Susceptibility Testing [43], if available for *Campylobacter* spp. Otherwise, “elevated non-wildtype MICs” were considered based on EUCAST *Campylobacter* spp. MIC distributions and the data obtained in our study for kanamycin (Figure S1). For lincomycin, the “elevated non-wildtype MICs” were based on a previous publication [44]; furthermore, the “elevated non-wildtype MICs” were established based on data from this study for nourseothricin and spectinomycin (Figure S1). For quality assessment of EUCAMP2 plate format, *C. jejuni* strain DSM 4688 (DSMZ - German Collection of Microorganisms and Cell Cultures GmbH, Braunschweig, Germany) and *C. coli* strain 2012-70-443-2 (Technical University of Denmark, Lyngby, Denmark) were included, which displayed sensitive phenotypes.

**Table 1:** Epidemiological cut-off values (ECOFFs, if available) or “elevated non-wildtype MIC values” for evaluation of antibiotic susceptibility testing results of thermotolerant *Campylobacter* spp.

Antimicrobial	MIC [mg/L], resistant >, *C. jejuni*	MIC [mg/L], resistant >, *C. coli*	Reference
Ampicillin	16	16	ECOFF for *C.* spp. [43]
Chloramphenicol	16	16	ECOFF for *C.* spp. [43]
Ciprofloxacin	0.5	0.5	ECOFF for *C.* spp. [43]
Erythromycin	4	8	ECOFF for *C.* spp. [43]
Florfenicol	4	4	ECOFF for *C.* spp. [43]
Gentamicin	2	2	ECOFF for *C.* spp. [43]
Kanamycin	16	16	elevated non-wildtype MICs ([43]; Fig. S1)
Lincomycin	8	8	elevated non-wildtype MICs [44]
Nalidixic acid	16	16	ECOFF for *C.* spp. [43]
Nourseothricin	4	4	elevated non-wildtype MICs (Fig. S1)
Spectinomycin	64	64	elevated non-wildtype MICs (Fig. S1)
Streptomycin	4	4	ECOFF for *C.* spp. [43]
Tetracycline	1	2	ECOFF for *C.* spp. [43]

The correlation of phenotypic resistance against antimicrobials on custom plates and presence of each known AMR gene was experimentally tested by analyzing at least five additional isolates without the resistance marker as negative control. For the frequently observed *bla*_OXA_ genes, a portion of *bla*_OXA_ positive isolates (139/459) underwent susceptibility testing with ampicillin (Table S1).

### Whole genome sequence analysis

DNA for short-read sequencing was extracted using the PureLink Genomic DNA Mini Kit (Thermo Fisher Scientific, Waltham, MA, USA) according to the manufacturer’s protocol. For this purpose *Campylobacter* isolates were subcultured on ColbA for 20 ± 2 h under microaerobic atmosphere at 42°C and bacteria were harvested from 1 mL of resuspended bacteria at OD_600_ of 2 by centrifugation at 14,000 x g for 5 min. The cell pellet was either directly used for DNA extraction or stored at -20°C. DNA for long-read sequencing was extracted using the MagAttract HMW Genomic Extraction Kit (Qiagen N.V., Venlo, The Netherlands) following manufacturer’s instructions, except starting with a cell pellet derived from 1 mL of bacteria at an OD_600_ of 2 upon centrifugation, followed by incubation for 1.5 h at 56°C and 900 rpm of agitation. The quality of the DNA was evaluated by spectral analysis (NanoDrop Spectrophotometer, Thermo Fisher Scientific, Waltham, MA, USA) and the concentration was fluorimetrically quantified by Qubit 3.0 Fluorometer (dsDNA HS Assay Kit 0.2–100 ng; Thermo Fisher Scientific, Waltham, MA, USA). DNA extracts for long-read sequencing were analyzed with the 5200 Fragment Analyzer System (Agilent Technologies Corp., Santa Clara, CA, USA) using DNF-464 HS Large Fragment Kit (Agilent Technologies Corp., Santa Clara, CA, USA) to check for DNA degradation/RNA contamination as well as sufficient length (> 10,000 bp) of the DNA fragments. DNA libraries for short-read sequencing were prepared using the Illumina DNA Prep, (M) Tagmentation Kit according to the manufacturer’s instructions (Illumina, Inc., San Diego, CA, USA) but using half of the volume of all reagents. Paired-end sequencing was performed on the Illumina MiSeq benchtop sequencer using the MiSeq reagent kit v3 (600 cycles, Illumina, Inc., San Diego, CA, USA) or on the Illumina NextSeq 500 sequencer using the NextSeq 500/550 mid output kit v2.5 (300 cycles, Illumina, Inc., San Diego, CA, USA) with read lengths ranging between 2 × 149 and 2 × 301, respectively. DNA libraries for long-lead sequencing (Oxford Nanopore Technology (ONT)) were prepared using the Rapid Barcoding Kit 96 (SQK-RBK110.96, Oxford Nanopore Technologies Limited, Oxford, United Kingdom) according to manufacturer’s instructions. Sequencing was performed on the MinION Mk1C instrument using a MinION FlowCell (R9.4.1, Oxford Nanopore Technologies Limited, Oxford, United Kingdom). For verification of truncation of the housekeeping multi-locus sequence typing (MLST) gene *aspA* in BfR-CA-16251, a PCR amplification of *aspA* was performed using the following primers, *aspA*-A9 (5’-AGT ACT AAT GAT GCT TAT CC-3’) and *aspA*-A10 (5’-ATT TCA TCA ATT TGT TCT TTG C-3’) [45; https://pubmlst.org/, last accessed on 05/01/2024]. Subsequently, the PCR fragment was purified using QIAquick PCR Purification Kit (Qiagen, N.V., Venlo, The Netherlands) and suitable amounts of DNA supplemented with either sequencing primer *aspA*-S3 (5’-CCA ACT GCA AGA TGC TGT ACC-3’) or *aspA*-S6 (5’-TTC ATT TGC GGT AAT ACC ATC-3’) [45; https://pubmlst.org, last accessed on 01/05/2024] were Sanger sequenced (Eurofins Scientific SE, Luxembourg City, Luxembourg).

### Bioinformatic Analysis

Illumina paired-end reads were trimmed and *de-novo* assembled with the AQUAMIS pipeline v1.3.8 [46], which implements e.g. fastp v0.23.2 for read quality control and trimming [47] and shovill v1.1.0 for assembly [48] as well as Quast v. 5.0.2 for assembly quality control. Sufficient quality was defined as base accuracy Q30 (error rate 1:1000) for more than 80% of the reads, and a minimum read coverage of 40. In addition, 10 sequences (Table S2) were also assembled using SKESA assembler using the NCBI Read Assembly and Annotation Pipeline Tool (RAPT at https://www.ncbi.nlm.nih.gov/rapt; last accessed on 01/05/2024).

Assembled contigs were analyzed for presence of resistance determinants as well as for plasmid markers using the BakCharak pipeline v3.0.3 [49]. The pipeline is composed of various modules, each serving a specific purpose. It includes the antimicrobial resistance gene finder module which identifies AMR determinants through the use of AMRFinderPlus v3.10.45 [50] and its corresponding AMRFinder database 2023-08-08.2. The Plasmidfinder employs ABRicate v1.0.1 [51] and utilizes the Center for Genomic and Epidemiology (CGE) plasmidfinder database. Default thresholds were applied for both ABRicate and AMRFinderPlus, which included a minimum identity threshold of 80% and 90%, respectively, and a minimum coverage threshold of 50% for both tools. Furthermore, Platon v1.6 [52] was used to predict putative plasmid location of contigs.

In addition to the BakCharak pipeline, assembled whole genome sequences from isolates showing pheno-genotype discrepancies were analyzed with ResFinder v4.1 [53] using low thresholds of identity (50%) and coverage (40%). This approach not only addressed missing genes in the AMRFinderPlus database but also revealed partial genes and those with reduced homology. Identified AMR gene sequences were extracted from the assembled sequences and analysed via the NCBI Basic Local Alignment Search Tool [54, 55] in order to find the closest AMR gene homolog. The latter search was conducted either using blastn or blastp, with the corresponding databases NCBI nucleotide collection (nr/nt) or non-redundant protein sequences (nr), respectively. Alignments of translated protein sequences were created using UniProt [56]. Subsequently the draft genome assemblies were screened with ABRicate v1.0.1 for their presence/absence of the respective AMR gene homolog (Table S3) using Linux command line. The reference resistance gene and protein sequences representing the most abundant closest relatives are depicted in Table S3. Alignments of nucleotide sequences and mapping of trimmed raw reads to reference resistance genes or the promoter region of *bla*_OXA_ genes was performed by Geneious Prime 2020.2.2 (Biomatters Ltd., New Zealand) using default settings. For verification of truncation of the housekeeping MLST gene *aspA* in BfR-CA-16251, *aspA* reference gene Cj0087 of *C. jejuni* NCTC 11168 (NC_002163.1) was used for mapping of raw reads and additional Sanger sequences were analyzed using SeqMan Pro (Lasergene 17, DNASTAR Inc., WI, USA).

Ridom SeqSphere + v8.4.2 (Ridom, Muenster, Germany) was used to perform phylogenetic analysis on assembled genome contigs from short-read sequencing using either the seven housekeeping genes based MLST or the core genome (cgMLST) scheme of 1343 gene targets previously defined [57]. A threshold of 98% identity and 98% of coverage to one of the respective alleles of the reference sequence NC_002163.1.gb (*C. jejuni* NCTC 11168) was used. At least 95% “good targets” were found based on cgMLST analysis. In addition, the 7-genes MLST scheme was used to lower the resolution for visualization of isolate diversity [45, https://pubmlst.org]. New MLST alleles and MLST sequence types were uploaded to PubMLST [58].

The Oxford Nanopore Technology sequencing data was basecalled using Guppy v. 6.0.1 in the “super-accuracy” mode (Oxford Nanopore Technologies, Oxford, UK). Subsequently, ONT reads were assembled and quality was assessed with the MiLongA Pipeline v1.0.1. [59]. This pipeline includes various tools, such as porechop v0.2.4 [60] for trimming and Unicycler v0.4.8 [61] for hybrid assembly. Assembled hybrid genome contigs from short- and long-read sequencing were annotated with Bakta [62] and AMR determinant identification was performed using AMRFinderPlus v3.10.45 [50] and its corresponding database (v. 2023-08-08.2). Raw read sequences and either complete genomes (for those isolates sequenced by ONT) or draft genomes were published within the BioProjects No. PRJNA562653, PRJNA595957, PRJNA648048 and PRJNA872862 at the NCBI sequence read archive (SRA) and Genome database.

### Statistical analyses

*Campylobacter* isolates were categorized into susceptible and resistant using the ECOFFs or elevated non-wildtype MIC values (Table 1). A variable “3–4 resistances” was defined for isolates with three or four resistances based on EUCAMP2 plate format, with nalidixic acid and ciprofloxacin being combined as (fluoro-)quinolones. An odds ratio (OR) with 95% confidence interval (CI) was calculated (Table S4, [63, 64]). *p*-values of less than 0.05 were considered statistically significant.

## Results

The 240 *C. coli*_*DE*_ (*n* = 115) and *C. jejuni*_*DE*_ (*n* = 125) isolates from Germany were taken from the strain collection of the laboratory. They were isolated from different matrices and locations in Germany by federal state laboratories as part of the zoonosis monitoring programs (Table S1, [12, 65]). The 254 *C. coli*_*VN*_ (*n* = 99) and *C. jejuni*_*VN*_ (*n* = 155) isolates from Vietnam were derived from fresh chicken fecal samples collected between November 2016 and December 2017 from primary chicken production of 26 different chicken farms and from cecum and retail samples in 2018 in the province of Hanoi and Haiphong. The principle size of the farms varied from 100 to 5,000 animals per flock, with only a few samples taken from farms with a flock size of 50,000. When farmers were asked for use of antimicrobials for treatment of chicken during rearing, they reported application of various substances, mostly tetracyclines (chlortetracycline, doxycycline and oxytetracycline), the macrolide tylosin, colistin as polymyxin, the ß-lactam amoxicillin and aminoglycosides like gentamicin and neomycin. In total, 254 Vietnamese thermotolerant *Campylobacter* spp. isolates were obtained, of which 155 were identified as *C. jejuni* and 99 as *C. coli*.

### Identification of highly resistant isolates using a standardized microtiter panel

**Fig. 1:**
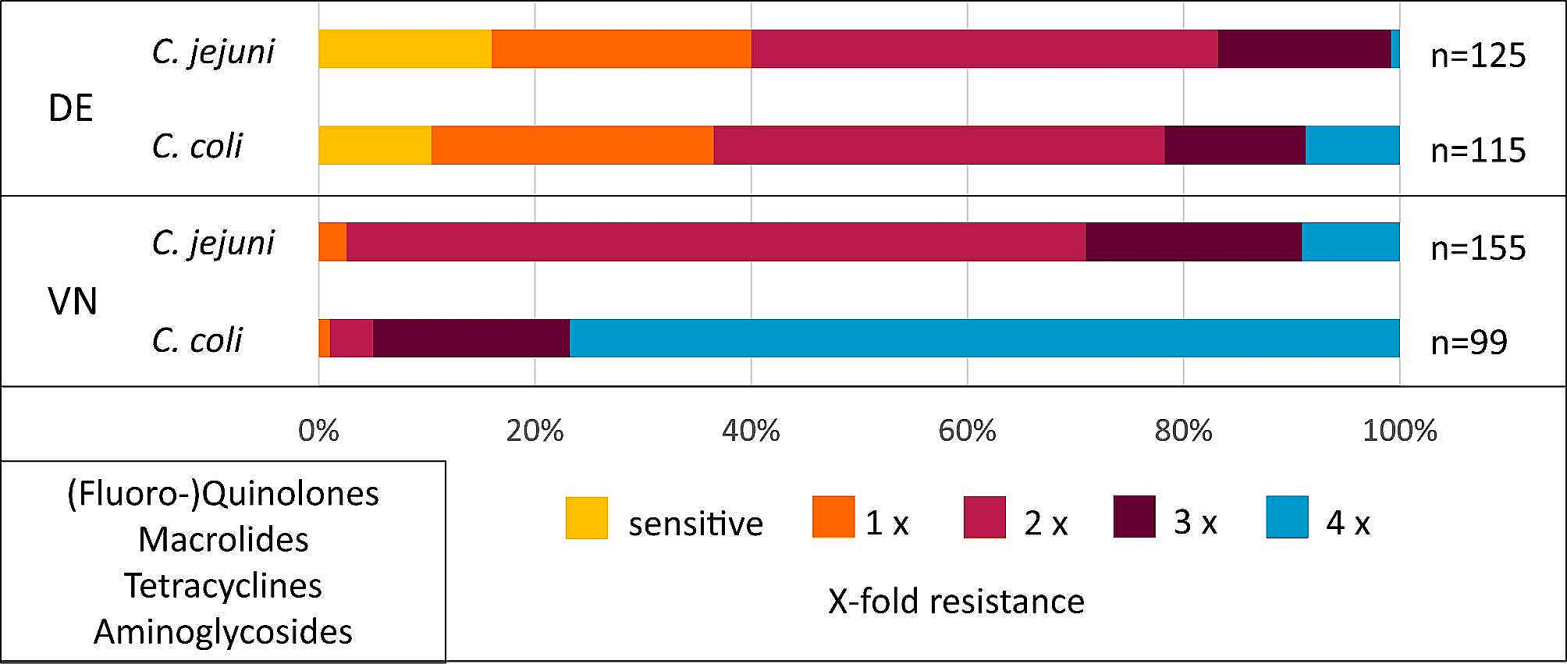
Vietnamese *C. coli* isolates displayed highest prevalence of combined resistance to all tested antimicrobial classes. Susceptibility to (fluoro-)quinolones (nalidixic acid, ciprofloxacin), macrolides (erythromycin), tetracycline and aminoglycosides (gentamicin, streptomycin) was tested by microdilution. X-fold resistance, number of antimicrobial classes to which isolates showed resistance (depicted in % of total number of tested isolates per category (n)); DE, German isolates; VN, Vietnamese isolates. Odds ratios are depicted in Table S4

Antimicrobial susceptibility testing was performed for all 494 isolates using the European standardized microtiter plate format EUCAMP2. The panel includes six representative antibiotics from four different antibiotic classes: aminoglycosides, (fluoro-)quinolones, macrolides, and tetracyclines. The proportion of sensitive isolates among the isolates from Germany was 10.4% (*n* = 12) for *C. coli* and 16% (*n* = 20) for *C. jejuni*; meanwhile, no sensitive isolates were detected among the *Campylobacter* spp. isolates from Vietnam (Fig. 1). In particular, 94.9% (94/99) of *C. coli*_*VN*_ and 29% (45/155) of *C. jejuni*_*VN*_ isolates were resistant to three or four compound classes. In comparison, *C. coli*_*DE*_ and *C. jejuni*_*DE*_ isolates were less frequently resistant to three or four compound classes (21.7%, 25/115 and 16.8%, 21/125, respectively).

Table 2 provides an overview of the prevalence of resistance to individual antimicrobials tested within the EUCAMP2 plate format. Phenotypic resistance to ciprofloxacin was high in *C. jejuni*_*DE*_ and *C. coli*_*DE*_ isolates (78.4 and 80.9%, respectively) whereas 98.1% of *C. jejuni*_*VN*_ isolates and all *C. coli*_*VN*_ displayed resistance to ciprofloxacin, respectively. Resistance to the erythromycin was low among *C. jejuni*_*DE*_ isolates, with only one resistant *C. jejuni*_*DE*_ isolate identified (0.8%) and moderately frequent among *C. coli*_*DE*_ isolates (18.3%).

**Table 2:** Antimicrobial resistance in *C. coli* and *C. jejuni* from Germany and Vietnam according to EUCAMP2 plates

	*Campylobacter jejuni*				*Campylobacter coli*			
	Germany (*n* = 125)		Vietnam (*n* = 155)		Germany (*n* = 115)		Vietnam (*n* = 99)	
	n	%	n	%	n	%	n	%
Ciprofloxacin	98	78.4	152	98.1	93	80.9	99	100.0
Erythromycin	1	0.8	17	11.0	21	18.3	76	76.8
Gentamicin	0	0.0	34	21.9	2	1.7	78	78.8
Nalidixic acid	92	73.6	149	96.1	92	80.0	99	100.0
Streptomycin	23	18.4	20	12.9	15	13.0	85	85.9
Tetracycline	80	64.0	154	99.4	80	69.6	98	99.0

ECOFFs (if available) or elevated non-wildtype MICs for resistance evaluation are depicted in Table 1; n, number of tested isolates; odds ratios are depicted in Table S4

In Vietnam, resistance to erythromycin was predominantly found for *C. coli*_*VN*_ isolates (76.8%), while 11% of the *C. jejuni*_*VN*_ isolates showed resistance to this antimicrobial substance. About two-third of *Campylobacter* isolates from Germany were tetracycline resistant (64 and 69.6% for *C. jejuni*_*DE*_ and *C. coli*_*DE*_, respectively). In comparison, the counterparts from Vietnam were almost completely resistant to this antibiotic (≥ 99%); in fact, only one *C. jejuni*_*VN*_ and one *C. coli*_*VN*_ isolate analyzed in this study were tetracycline sensitive. Resistance to gentamicin was only detected in two *C. coli*_*DE*_ isolates, whereas all *C. jejuni*_*DE*_ were sensitive. In contrast, 78.8% of the *C. coli*_*VN*_ isolates and 21.9% of the *C. jejuni*_*VN*_ were resistant to gentamicin. Resistance to streptomycin was highest in *C. coli*_*VN*_ isolates (85.9%), while in *C. coli*_*DE*_ this resistance was moderately frequent with 13%, which was similar to *C. jejuni*_*VN*_ (12.9%). The *C. jejuni*_*DE*_ isolates were slightly more resistant to streptomycin (18.1%) than the *C. coli*_*DE*_ isolates and the *C. jejuni*_*VN*_ isolates but this was not statistically significant. Overall, the isolates from Vietnam were 5.1 times more likely resistant to three or more antibiotics compared to their counterparts from Germany (OR 5.1, 95% CI 3.4–7.6; Table S4). Taking the same variable of “3–4 resistances”, *C. coli* isolates from Vietnam were far more resistant against the tested antimicrobials than the *C. jejuni* isolates from the same geographic location (OR 46.0, 95% CI 17.5-120.5). The likeliness of displaying 3–4 resistances was not significantly different for *C. coli*_*DE*_ versus *C. jejuni*_*DE*_ (*p* = 0.33). However, significantly different acquisition of resistance to erythromycin was observed for *C. coli* isolates compared to *C. jejuni* not only in Vietnam (OR 26.8, 95% CI 13.5–53.3) but also in Germany (OR 27.7, 95% CI 3.7-209.7).

### Phylogenetic diversity of strains is a good basis for in-depth AMR analysis

**Fig. 2:**
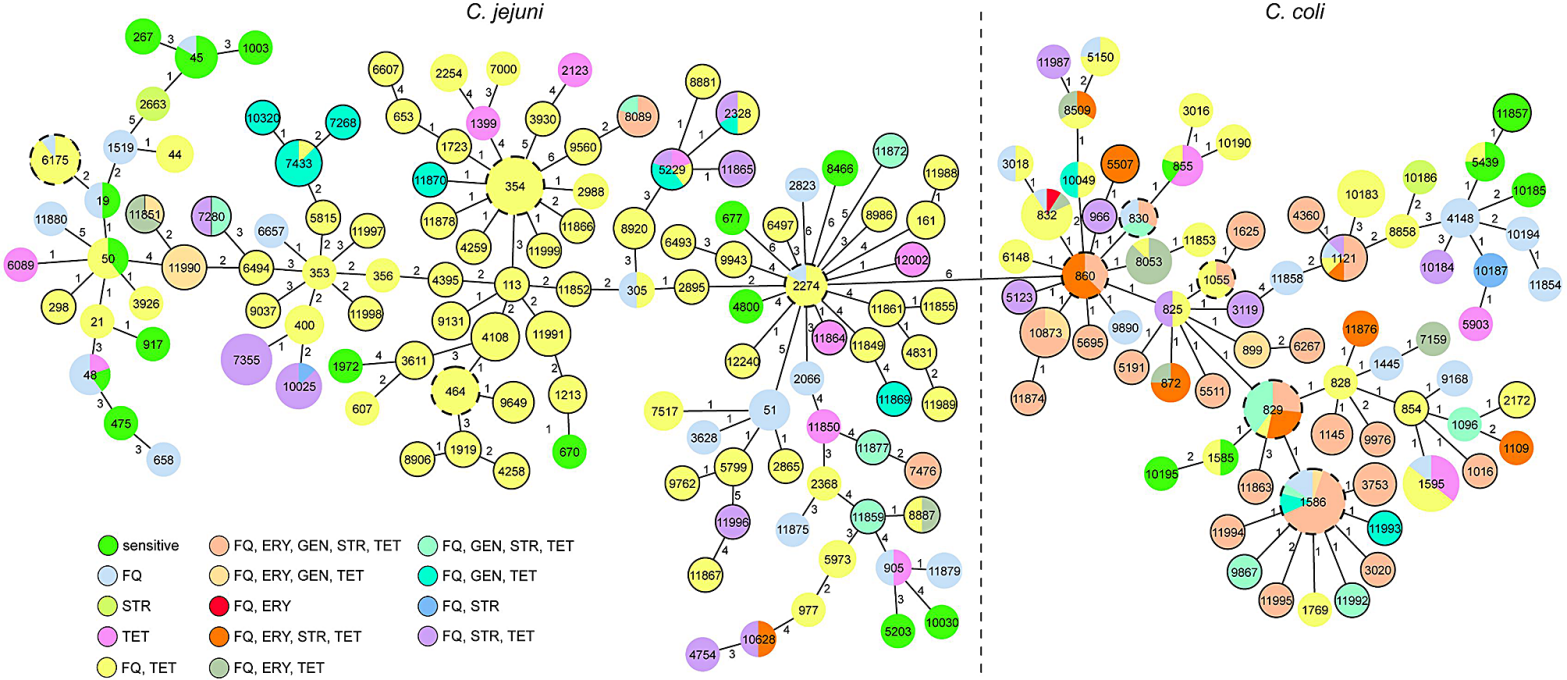
Test strains showed phylogenetical diversity, with AMR patterns distributed all over the identified MLST types. Minimum spanning tree (MST) based on MLST analysis. Colors indicate different phenotypic resistance profiles obtained with EUCAMP2 plate format. Nodes with numbers represent ST types; node size corresponds to the number of isolates (e.g. ST-267 is only represented by one isolate). Closed circles, Vietnamese isolates; open circles, German isolates; dashed-lined circles, isolates from both countries. FQ, (fluoro-)quinolone resistant; STR, streptomycin resistant; ERY, erythromycin resistant; TET, tetracycline resistant; GEN, gentamicin resistant. Numbers between nodes indicate numbers of allele difference based on 7 housekeeping genes (cgMLST differences are depicted in Table S2). MST was created with Ridom SeqSphere + software.

All 494 isolates were subjected to whole-genome sequencing using short-read Illumina technology. To determine phylogenetic relationship of the *Campylobacter* isolates, first multi-locus sequence typing method (MLST) for comparison of the seven housekeeping genes was applied (Fig. 2). For higher resolution, the core gene MLST (cgMLST) scheme based on the comparison of 1343 core genes was used [57] with missing loci pairwise ignored (Ridom SeqSphere+) (Table S5). We identified 15 new MLST allele variants, including an *aspA* allele with a deletion of 19 bases in BfR-CA-16251 (Figure S2) and assigned 191 different sequence types (STs), of which 41 were novel (Fig. 2, Table S1). The *C. jejuni*_*VN*_ subpopulation possessed the greatest diversity of different ST types (*n* = 70), followed by the *C. jejuni*_*DE*_ subpopulation (*n* = 53). *C. coli* possessed less diversity, since isolates from Germany belonged to 45 different STs, while *C. coli* from Vietnam were attributed to 32 different STs. They were part of the common clonal complexes CC-828 (*n* = 148) or CC-1150 (*n* = 15) or did not belong to any CC (*n* = 51). Although, some isolates from Germany and Vietnam shared the same MLST sequence types (n_ST_=9, Fig. 2, circles with dashed line), they were not phylogenetically related on the basis of cgMLST (Table S5). Consistently, resistance patterns were independent of phylogenetic origin, since similar AMR patterns were distributed all over the identified MLST types (Fig. 2).

### Distribution of resistant determinants in *Campylobacter* spp. from Germany and Vietnam

**Fig. 3:**
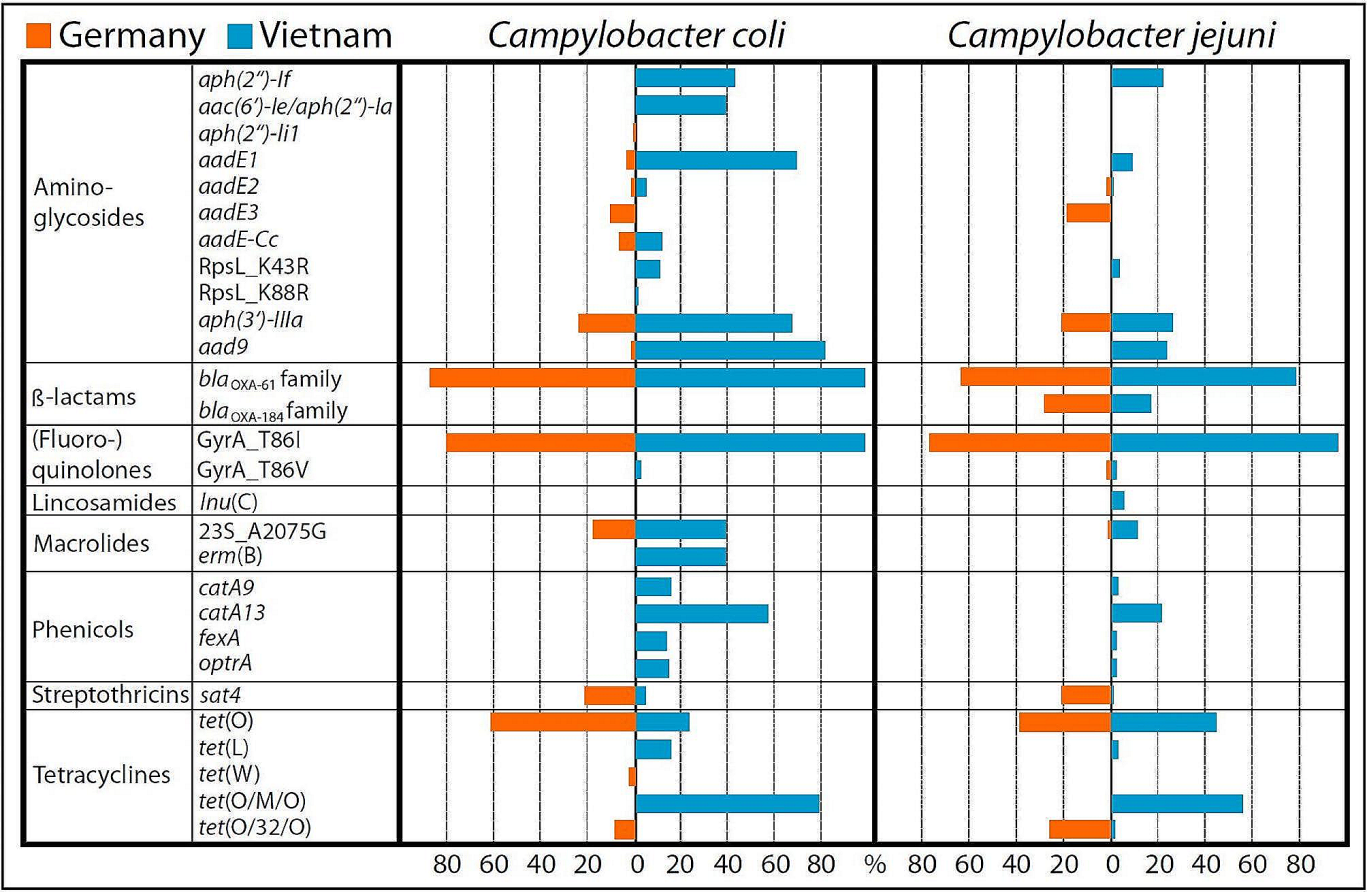
Distribution and prevalence of resistance determinants identified by whole genome sequencing in *Campylobacter* spp. Fraction (%) of German (orange bars) and Vietnamese (blue bars) *C. jejuni* (*n* = 280) and *C. coli* (*n* = 214) isolates, carrying the respective resistance determinant are depicted. Resistance determinants are sorted according to antibiotic class. Partial and full-length genes are considered

Short-read whole genome sequencing results were processed using the AMRFinderPlus tool [50] for identification of AMR genes. In total, 22 different resistance genes and gene variants (e. g. *erm*(B), *tet*(O), *aadE*, *aph(3’)-IIIa*, *aad9*, *catA*, *lnu*(C), *bla*_OXA_, *sat4*) and point mutations in three distinct genes (*gyrA*, 23S rRNA, *rpsL*) associated with AMR were identified (Fig. 3 and Table S1). The resistance determinants were differently distributed among *Campylobacter* populations from Germany and Vietnam and fewer AMR genes were found in *C. jejuni* compared to *C. coli* (Fig. 3). We first checked whether the identified genes could be associated with the phenotype obtained by the EUCAMP2 plate format (Table 2). In case other resistance genes were identified via WGS analysis, custom plate microdilution for characterization of antimicrobial susceptibility was performed. Hence, the expected phenotypic resistance based on the presence of each AMR gene was experimentally tested. Table 3 summarizes the concordances and discrepancies of phenotypic and genotypic resistance characteristics of the isolates sorted by antibiotic class (detailed in Table S1), which we address in the following sections. As proof of principle, a selection of 14 isolates was also subjected to long-read ONT sequence analysis.

**Table 3:** Correlation and discrepancies between genotype and phenotype of *Campylobacter* spp. resistance profile using AMR FinderPlus

Antibiotic class	Antibiotic tested	#Isolates tested	#Isolates resistant	Resistance determinant	Correlation between pheno- and genotype	Reason for discrepancy†
Aminoglycosides	GEN	494	114	*aph(2’’)-li*_*1*_, *aph(2’’)-If*, bifunctional *aac(6’)-Ie*/*aph(2’’)-Ia*	99.1% (113/114)	isolate with yet unknown resistance determinant (MIC > 16 mg/L; GEN-R; *n* = 1),
	STR	494	143	*aadE1-3*, *aadE-Cc*, RpsL_K43R, RpsL_K88R	78.1% (114/146)	*aadE*3 gene not found (missing in database (WP_057035408.1); STR-R; *n* = 29), partial *aadE*1 genes (STR-R; *n* = 2), isolate slightly resistant (MIC = 8 mg/L), but harboring none of the known resistance determinants (*n* = 1)
	KAN	157	139	*aph(2’’)-If*, bifunctional *aac(6’)-Ie*/*aph(2’’)-Ia*, *aph(3’)-IIIa*	100% (139/139)	n.a.
	SPC	139	118	*aad9*	30.5% (36/118)	partial genes found due to frame-shift within poly-C tract (SPC-R; *n* = 82)
β-Lactams	AMP	139	134	*bla*_OXA−61_ and _− 184_ family genes	98.5% (132/134)	Isolate susceptible (MIC = 16 mg/L), but harboring *bla*_OXA−193_ (*n* = 1); isolate slightly resistant (MIC = 32 mg/L), but harboring no *bla*_OXA_ gene (*n* = 1)
(Fluoro-) Quinolones	CIP	494	442	GyrA_T86I, GyrA_T86V	100% (442/442)	n.a.
	NAL	494	432	GyrA_T86I, GyrA_T86V	97.7% (432/442)	CIP-R/NAL-S phenotype, yet unknown mechanism (*n* = 10)
Lincosamides	LCM	44	35	*lnu*(C), 23S_A2075G, *erm*(B)	88.6% (31/35)	isolates slightly resistant (MIC = 16 mg/L), but harboring none of the known resistance determinants (*n* = 4)
Macrolides	ERY	494	115	23S_A2075G, *erm*(B)	100% (115/115)	n.a.
				50S_L22_A103V	20% (31/155)	point mutation not associated with resistance to erythromycin; 20% correlation due to additional presence of either 23_A2075G or *erm*(B)
Nourseothricin	NTC	98	56**	*sat4*	100% (56/56)	n.a.
Phenicols	CHL	130	93	*catA9*, *catA13*, *fexA*, *optrA*	100% (94/94)	n.a.
	FLO	53	29	*fexA*, *optrA*	62.1% (18/29)	isolates slightly resistant (MIC = 8–16 mg/L) in absence of known phenicol resistance determinants (*n* = 11)
Tetracyclines	TET	494	412	*tet*(O), *tet*(O/32/O), *tet*(W), *tet*(O/M/O), *tet*(L)	84.6% (351*/415)	partial (mosaic-) *tet*(O) genes found (TET-R; *n* = 54), (mosaic-) *tet*(O) genes not found, but phenotypic resistance expressed (under coverage threshold; TET-R; *n* = 8), *tet*(W) with two point mutations (G511A/G1736A leading to D171N/G579D; TET-S; *n* = 2)

AMP, ampicillin; CHL, chloramphenicol; CIP, ciprofloxacin; ERY, erythromycin; FLO, florfenicol; GEN, gentamicin; KAN, kanamycin; LCM, lincomycin; NAL, nalidixic acid; NTC, nourseothricin; SPC, spectinomycin; STR, streptomycin; TET, tetracycline; n.a., not applicable (100% correlation); * *tet*(O/32/O) found as *tet*(O) with reduced identity (93.4%; missing in database; TET-R; *n* = 41) not depicted here, since pheno- and genotype were consistent with only incorrect nomenclature; **Considering a cut-off value of > 4 mg/L as resistant; †Based on the prediction of resistance determinants obtained via the BakCharak pipeline (comprises the AMRFinderPlus tool and its corresponding database version 2023-08-08.2)

### Resistance to (fluoro-)quinolones

The mutation T86I in the gyrase A subunit was the most prominent mutation found to be associated with resistance to (fluoro-)quinolones. The T86I was found in 98.4% (*n* = 436), while the T86V mutation was identified in only 1.6% (*n* = 7) of the ciprofloxacin resistant isolates. Nearly all (97%, *n* = 246) isolates from Vietnam and 79% (*n* = 189) of the isolates from Germany contained this specific resistance mechanism (Fig. 3). Only five isolates from Vietnam and two from Germany showed the mutation T86V and displayed resistance to nalidixic acid and ciprofloxacin. Three isolates from Vietnam and seven isolates from Germany with the T86I mutation in GyrA were resistant to ciprofloxacin (MIC_CIP_ = 4–16 mg/L) but sensitive to nalidixic acid. Interestingly, six of the seven isolates from Germany susceptible to nalidixic acid had MIC values ≤ 2 mg/L, while being resistant to ciprofloxacin.

### Resistance to macrolides and lincosamides

In all macrolide resistant isolates from Germany (*n* = 22/22) and in 59% of the isolates from Vietnam (*n* = 55/93) the single point mutation A2075G in the 23S rRNA gene was found, conveying erythromycin resistance. However, 38 *C. coli*_*VN*_ isolates harbored the gene *erm*(B), encoding an rRNA adenine N-6-methyltransferase, modifying the target binding site for macrolides in the 23S rRNA, thus conferring resistance to macrolides [66]. The MIC distribution of isolates carrying the 23S rRNA A2075G point mutation or the *erm*(B) gene was comparable, ranging from 64 mg/L (n_*ermB*_ = 6; n_23S_A2075G_ = 5) to 128 mg/L (n_*ermB*_ = 5; n_23S_A2075G_ = 4) and exceeding 128 mg/L (n_*ermB*_ = 27; n_23S_A2075G_ = 68) (Figure S3). The translated *erm*(B) genes shared 99 − 100% amino acid identity to the reference Erm(B) protein WP_002321849.1, with maximally one conservative mutation (I125V) in three *C. coli* isolates from Vietnam (BfR-CA-16073, BfR-CA-16297, BfR-CA-18879), displaying MIC values > 128 mg/L to erythromycin. The resistance gene *lnu*(C), which codes for a lincosamide nucleotidyltransferase (100% identity shared with WP_002837187.1) was found in eight *C. jejuni* isolates from Vietnam. In four of the eight isolates, the point mutation A2075G in 23S rRNA was also present, which is sufficient for resistance to lincomycin. However, the other four isolates, harboring the *lnu*(C) gene in absence of the 23S rRNA A2075G point mutation, were sensitive to erythromycin but resistant to lincomycin (MIC of 64 to > 128 mg/L), indicating *lnu*(C) as the determinant for lincomycin resistance in these isolates. The point mutation A103V in the L22 ribosomal protein was identified in 124 macrolide sensitive isolates, from which 103 isolates displayed MICs of ≤ 1 mg/L erythromycin. Furthermore, the point mutation was identified in three lincomycin sensitive isolates. Hence, this mutation alone did not confer resistance to macrolides nor lincomycin.

### Resistance to tetracyclines

**Fig. 4:**
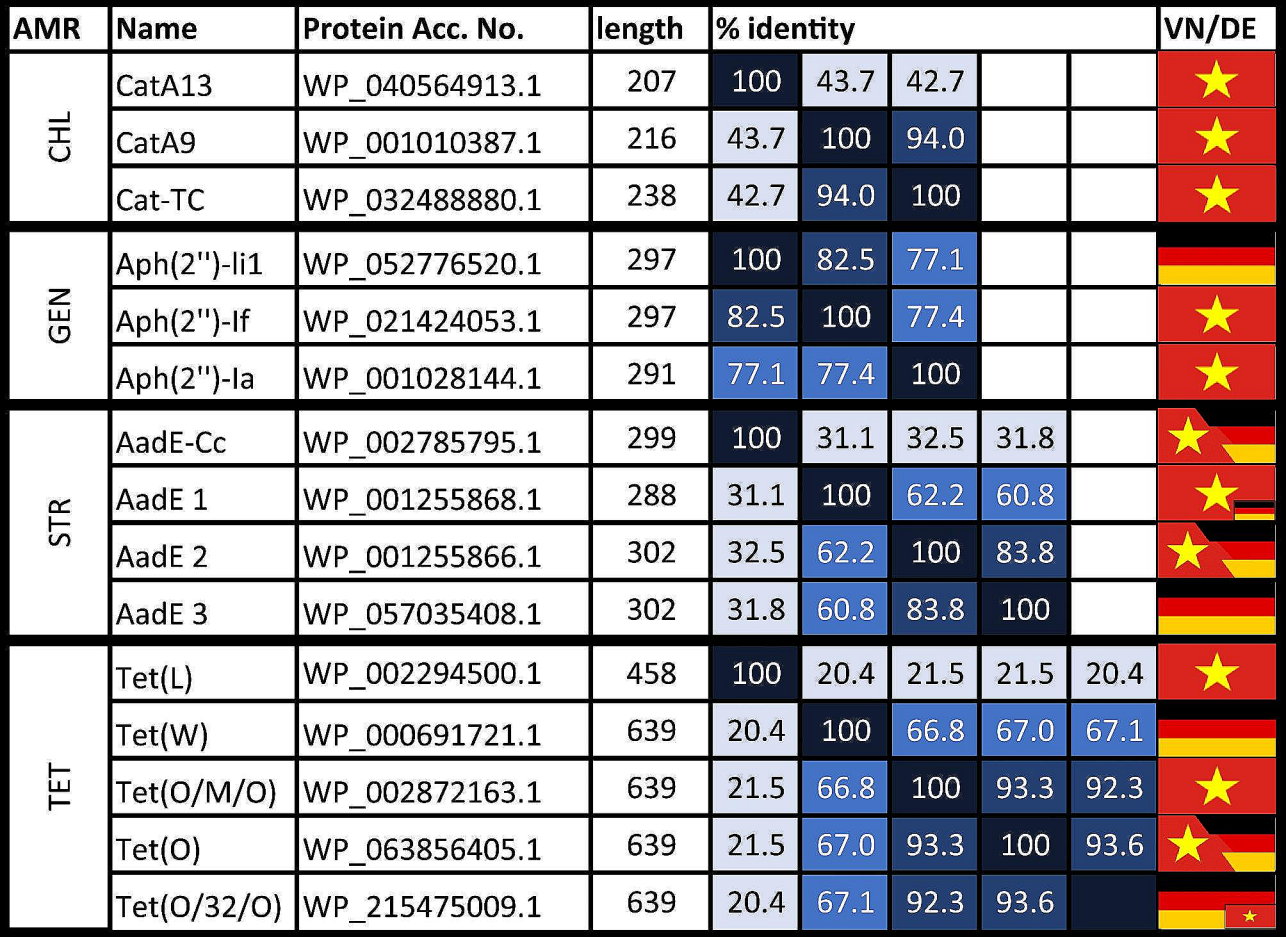
Visualization of the protein variants found in thermotolerant *Campylobacter* spp. Closest NCBI matches with accession number (Acc. No.), amino acid length and percentage of protein identity to each other are depicted (computed with UniProt Align tool (Release 2023_02, [56])). Country-specific prevalence is highlighted with national flags, whose sizes correspond to the magnitude of prevalence (detailed in Fig. 3). CHL, chloramphenicol; GEN, gentamicin; STR, streptomycin; TET, tetracycline; VN, Vietnam; DE, Germany. Percent sequence identity is colored as follows: 100%, black; 80–99%, dark blue; 60–79%, blue; ≤ 59%, light blue

Tetracycline resistance of *Campylobacter* isolates was associated with the presence of either the ribosomal protective protein-encoding genes *tet*(O), mosaic variants (*tet*(O/M/O), *tet*(O/32/O), the latter missing in the AMRFinder database) or *tet*(W), or the efflux transporter-encoding gene *tet*(L). ResFinder enabled the detection of the Tet(O) protein variants, which share ≥ 92.3% identity with each other, while Tet(W) shows ~ 67% identity to the Tet(O) proteins (Fig. 4).

In some resistant isolates, *tet*(O) and/or its mosaic variants were either partially found at the end of contigs (*n* = 54) or were falsely absent (*n* = 8). These isolates were reanalyzed by mapping raw reads to reference tetracycline resistance genes using Geneious Prime software (exampled in Figure S4). As a result the presence of multiple different variants of *tet*(O), including isolates exhibiting unique variants of *tet*(O/M/O) with varying degree and localisation of *tet*(M) sequence introgression, could be detected (Figure S5, visualized using [67], Table S1). However, as expected, mapping of reads to template *tet*(O) gene variants did not provide information about the presence of multiple identical full-length and/or partial gene copies. We confirmed the presence of multiple copies of identical or *tet*(O) variants by employing AMRFinderPlus on hybrid assemblies obtained from long-read sequencing of selected isolates. Consistently, except for one isolate (BfR-CA-17105), only long-read sequencing was capable of identification of multiple identical copies of *tet*(O) genes. Long-read sequencing also detected different truncated versions of *tet*(O) variants (in BfR-CA-15991, BfR-CA-18842, BfR-CA-16077, BfR-CA-16088, BfR-CA-16297 and BfR-CA-19087) or a mutated *tet*(O) leading to a premature stop codon and a truncated protein (BfR-CA-16040) (Table 4). Those seven isolates with tetracycline resistance also harbored one or two additional copies of *tet*(O) or gene variants.

**Table 4:** Long-read sequencing found AMR genes often near transposase genes and on the chromosome

Isolate No.	VN/DE	Spec	predictions short-read assembly				predictions based on ONT/hybrid assembly			
			plasmids contigs	plasmids circular	plasmids mobilization elements	plasmids conjugation elements	circular contigs	plasmid contigs	Point mutations and AMR genes on chromosome (in bold indicates location in proximity to transposase genes)	AMR genes on plasmid
BfR-CA-15687	DE	Cc	**1** (-)	0	**1**	**6**	2	1	*bla*_OXA_-489;*tet*(O);GyrA_T86I	*tet*(O)
BfR-CA-15991	VN	Cc	**0**	**0**	**0**	**0**	1	0	***tet*(O/M/O)-*catA9*-*tnp***_**IS1216 family**_**-*fexA*-*optrA*-*tnp***_**IS1216family**_**-*tet*(L)**;*aac(6’)-Ie/aph(2’’)-Ia*-*aadE*1-*tet*(O)x_ΔC−terminus_*bla*_OXA_-193;23S_A2075G; 23S_A2075G; 23S_A2075G;GyrA_T86I	
BfR-CA-16040	VN	Cc	1 (-)	**0**	**0**	**0**	1	0	***tnp***_**ISCco2 family**_***-catA13*-*aph(3’)-IIIa*-*aad9***;*tet*(O/M/O)-*aad9*-*erm*(B)-*aadE*1;*aac(6’)-Ie/aph(2’‘)-Ia*-*aadE*1-*tet*(O)x_ΔC−terminus_; *bla*_OXA_-489;GyrA_T86I	
BfR-CA-16046	VN	Cc	**2** (-)	**1**	**1**	**6**	2	1	***tnp***_**ISCco2 family**_***-catA13*-*aph(3’)-IIIa*-*aad9*-*aph(2’‘)-If*-*bla***_**OXA**_**-193**;*tet*(O/M/O)-*aad9*-*erm*(B)-*aadE*1;*aadE-Cc*;GyrA_T86I	
BfR-CA-16077	VN	Cj	1 (-)	**0**	**0**	**0**	1	0	***tnp***_**ISCco2 family**_***-catA13*-*aph(3’)-IIIa*-*aad9*-*aph(2’‘)-If***;*tet*(O/M/O)-*aadE*1-*tet*(O)x_ΔN−terminus_;***aph(3’)-IIIa-tnp***_**ISCaje6 family**_;*bla*_OXA_-184 family; ***tnp***_**ISCco2 family**_**-*lnu*(C)**;GyrA_T86I	
BfR-CA-16088	VN	Cj	1 (-)	**0**	**0**	**0**	1	0	***tnp***_**ISCco2 family**_***-catA13*-*aph(3’)-IIIa*-*aad9*-*aph(2’‘)-If***;*tet*(O/M/O)-*aadE*1-*tet*(O)x_ΔN−terminus_;***aph(3’)-IIIa-tnp***_**ISCaje6 family**_;*bla*_OXA_-184 family; ***tnp***_**ISCco2 family**_**-*lnu*(C)**;GyrA_T86I;23S_A2075G;23S_A2075G;23S_A2075G	
BfR-CA-16110	VN	Cc	**0**	**0**	**0**	**0**	1	0	*tet*(O)-*aad9*-*erm*(B)-*aadE*1;*bla*_OXA_-193;GyrA_T86I	
BfR-CA-16201	VN	Cc	0	0	0	0	2	1	*tet*(O)-*aad9*-*erm*(B)-*aadE*1; ***tnp***_**ISCco2 family**_***-catA13*-*aph(3’)-IIIa*-*aad9*-*aph(2’‘)If*-*bla***_**OXA**_**-193**;*tet*(O);GyrA_T86I	
BfR-CA-16258	VN	Cc	**1** (+)	**1**	0	0	2	1	*tet*(O)-*aad9*-*erm*(B)-*aadE*1;*tet*(O);*bla*_OXA_-193;GyrA_T86I	
BfR-CA-16297	VN	Cc	**0**	**0**	**0**	**0**	1	0	*tet*(O/M/O)-*aad9*-*erm*(B)-*aadE*1;***aph(2’‘)-If*-*aph(3’)IIIa-tnp***_**ISCaje6 family**_; *aac(6’)-Ie/aph(2’’)-Ia*-*aadE*1-*tet*(O)x_ΔC−terminus_;***tet*(O)-*tnp***_**IS607 family**_;*bla*_OXA_-193;GyrA_T86I	
BfR-CA-16737	DE	Cj	**2** (+)	0	**1**	**6**	2	1	*bla*_OXA_-185 like;*tet*(O);GyrA_T86I	*tet*(O/32/O)-*aadE*2_Δ1-415-*sat4*-*aph(3’)-IIIa*
BfR-CA-18842	VN	Cc	**0**	**0**	**0**	**0**	1	0	*aac(6’)-Ie/aph(2’’)-Ia*-*aadE*1-*tet*(O)x_ΔC−terminus_; *tet*(O/M/O);*bla*_OXA_-193;GyrA_T86I	
BfR-CA-19087	DE	Cc	**3** (+)	0	0	0	2	1	***tnp***_**IS607 family**_***-tet*(O/32/O)-*aph(2’‘)-li***_***1***_**-*aph(3’)-IIIa*-*aad9*-*aadE*1-*tet*(O)**x_ΔN−terminus_;*tet*(O/32/O);*aadE-Cc*;GyrA_T86I	
BfR-CA-19301	VN	Cj	**0**	**0**	**0**	**0**	1	0	***aadE*3-*sat4*-*aph(3’)IIIa-tnp***_**IS1216 family**_;*tet*(O/32/O);*bla*_OXA_-193; GyrA_T86I	

Bold numbers, plasmid predictions based on short-read sequence data are consistent with ONT data; (+), true; (-), false prediction of AMR gene localization on plasmids compared to ONT data. Genes in bold depict AMR determinants located on the chromosome in proximity to transposase genes. 50S_L22_A103V mutation was omitted due to absence of resistance phenotype; VN, Vietnam; DE, Germany; Spec, Species; Cj, *C. jejuni*; Cc, *C. coli*

Within the tested concentration range (0.5–64 mg/L), we did not find differences in the degree of resistance associated with a single copy of *tet*(O) or its variant genes or with multiple copies of *tet* genes. The predominant resistance gene (full-length and/or partial) among the isolates tested was *tet*(O) (119 and 91 isolates from Germany and Vietnam, respectively). *tet*(O/M/O) was exclusively found in isolates from Vietnam (*n* = 164) and *tet*(O/32/O) predominantly in isolates from Germany (n_DE_=42, n_VN_=2). Thus, different *Campylobacter* populations harbored distinct gene variants. One of the *C. coli*_*DE*_ isolates (BfR-CA-17078) carrying the *tet*(O/32/O) gene was sensitive to tetracycline and carried a point mutation introducing a stop codon (G1475A; p.W492Ter). The correlation of *tet*(L) presence and tetracycline resistance in *Campylobacter* was only shown in isolates also carrying *tet*(O). Three isolates from Germany harbored the *tet*(W) gene, yet two of them were sensitive to tetracycline and showed the same amino acid substitutions (D171N and G579D) (Figure S6).

### Resistance to the aminoglycosides gentamicin and kanamycin

Gentamicin resistance in *Campylobacter* was rare in Germany, with only two identified resistant *C. coli*. One of the two isolates harbored the resistance gene *aph(2’’)-li*_*1*_, which encodes an aminoglycoside phosphotransferase [68]. For the second isolate, the genetic determinant for gentamicin resistance was not detected but phenotypic resistance was repeatedly observed by microdilution assays (MIC > 16 mg/L). Here, further studies are needed to decipher the underlying mechanism of gentamicin resistance. In 112 gentamicin resistant isolates from Vietnam, the aminoglycoside phosphotransferase gene *aph(2’’)-If* (*n* = 76) and the gene *aac(6’)-Ie/aph(2’’)-Ia* (*n* = 38) were found, the latter coding for a bifunctional enzyme combining a phosphotransferase with an N-acetyltransferase. Both resistance determinants also confer resistance to kanamycin.

Kanamycin resistance was further associated with the presence of the aminoglycoside phosphotransferase *aph(3’)-IIIa*. In total 160 isolates contained this gene (n_VN_=106, n_DE_=54) and were phenotypically resistant to kanamycin. Among them were 97 isolates (n_VN_=96, n_DE_=1) with a combination of *aph(3’)-IIIa and* either *aph(2’’)-If* (n_VN_=73) or the bifunctional gene (n_VN_=21), both conferring gentamicin and kanamycin resistance, or *aph(2’’)-li*_*1*_ (n_DE_=1). Furthermore, two additional *C. coli*_*VN*_ (BfR-CA-16297, BfR-CA-18728) harbored a combination of *aph(3’)-IIIa, aph(2’’)-If* and the bifunctional gene and, thus, acquired two genetic determinants redundantly encoding a gentamicin and kanamycin modifying enzyme and a further enzyme for kanamycin inactivation. Intriguingly, long-read sequencing even revealed two isolates (BfR-CA-16077, BfR-CA-16088) with two copies of *aph(3’)-IIIa* in combination with *aph(2’’)-If*. Within the test ranges of gentamicin (0.12–16 mg/L) and kanamycin (2–1024 mg/L), we could not observe increased MIC values for isolates containing multiple redundant resistance determinants compared to isolates only harboring a single gene.

### Resistance to the aminoglycoside streptomycin

Four variants of *aadE* genes (*aadE-Cc* and *aadE* 1, 2, 3, Fig. 4), coding for aminoglycoside 6-adenylyltransferases and two additional point mutations in the *rpsL* ribosomal gene were associated with streptomycin resistance in the *Campylobacter* spp. isolates. The predominant streptomycin resistance gene in Vietnam was *aadE*1 (WP_001255868.1, n_VN_=82, n_DE_=2). AMRFinderPlus identified a partial *aadE*1 gene (88.9% protein sequence coverage) in two of these isolates from Vietnam (BfR-CA-19112, BfR-CA-19119), which displayed resistance to streptomycin. Mapping of raw reads to reference gene *aadE*1 revealed the presence of the full-length gene, thus indicating an assembly error. Both isolates additionally carried a partial *aadE*2 (Δ1-109 bp) as verified by extraction of the Bakta annotated coding sequences and subsequent alignment to a reference gene (NG_047393.1, Table S3). This observation explained streptomycin resistance in these two isolates. Hence again, the presence of redundant homologous genes resulted in contig breaks during the assembly process, impeding the accurate reconstruction of genes from short-read sequences. In total, mapping of reads to template *aadE2* revealed eight isolates displaying truncated non-functional AadE2 (WP_001255866.1), among them three streptomycin sensitive isolates from Germany (BfR-CA-16737, BfR-CA-16834, BfR-CA-19311), confirming loss of function of AadE2 due to truncation (*aadE2*_Δ1-415). The five isolates from Vietnam also contained full length *aadE*1, consistent with their streptomycin resistant phenotype. The AadE3 variant (WP_057035408.1), exclusively found in isolates from Germany (*n* = 29), is missing in the AMRFinderPlus database and was, thus, only found by manual ABRicate search using the *aadE*3 reference nucleotide sequence (Table S3). The AadE-Cc variant (WP_002785795.1) was detected in *C. coli*_*VN*_ (*n* = 11) and *C. coli*_*DE*_ (*n* = 8). While three isolates from Vietnam and one from Germany in addition contained the *aadE*1, one isolate from Germany displayed streptomycin sensitivity, corresponding to a *aadE-Cc* with a point mutation (ΔA558; p.A187LfsTer188) leading to early termination of translation, correctly annotated by AMRFinderPlus.

A point mutation in the RpsL ribosomal protein was rare and only observed in isolates from Vietnam. The RpsL K43R point mutation was present in 10 *C. coli* and 5 *C. jejuni* isolates, while one *C. coli* harbored the RpsL K88R mutation (BfR-CA-18880). Isolates carrying either RpsL K43R or RpsL K88R were resistant to streptomycin (MIC > 16 mg/L). One of these isolates (BfR-CA-18738) additionally carried the *aadE*1 gene.

### Resistance to the aminoglycoside spectinomycin

Spectinomycin resistance was widespread among isolates from Vietnam (*n* = 116) and rare among isolates from Germany (*n* = 2). In our study, the presence of a gene encoding the spectinomycin adenyltransferase Aad9 (WP_002578722.1) was associated with high-level resistance (MIC of 256 to > 512 mg/L) and was carried by 80.8% *C. coli*_*VN*_ and 23.2% *C. jejuni*_*VN*_ isolates, as well as by the two *C. coli*_*DE*_ isolates (Fig. 3).

In the majority of spectinomycin resistant isolates (*n* = 82/118), the AMRFinderPlus identified the presence of a truncated version of *aad9* (69.8 to 88.0% gene coverage to WP_002578722.1). Again, this was partially due to an inability of correct identification of full-length *aad9* genes from short-read sequence data caused by the presence of multiple copies of *aad9*, confirmed by long-read sequencing (e.g. BfR-CA-16040 and BfR-CA-16046). Additionally, we observed frameshifts within a putative poly-C tract present in the resistance gene, leading to a truncated Aad9 protein. However, all isolates, carrying *aad9* showed phenotypic resistance. We wondered whether *aad9* inactivation by poly-C was only present in a subpopulation of the bacterial suspension and/or whether frame-shifting can lead to restoration of a full-length protein. Indeed, when we mapped raw reads to an *aad9 C. coli* reference gene linked to the reference protein WP_057031337.1 (Acc. NZ_CP091310.1:1,750,066–1,750,845), we detected a variable number of cytosines in the frame shift region in some of the sequences, suggesting that *aad9* undergoes phase variation. To identify potential reversal of the correct number of cytosines in the poly-C tract associated with phenotypic resistance, we subjected one of the isolates, BfR-CA-15987, to selection pressure on ColbA plates supplemented with 128 mg/L spectinomycin, followed by whole genome sequencing analysis. Analysis of sequence data before and after selection on spectinomycin showed that spectinomycin selected for BfR-CA-15987 clones with one additional cytosine within the poly-C tract, restoring the full-length gene (Figure S7).

### Resistance to chloramphenicol and florfenicol

Resistance to antibiotics from the phenicol group was only observed in the isolates from Vietnam. About 58% (*n* = 57 of the *C. coli* and 23% (*n* = 36) of the *C. jejuni* isolates carried one or multiple phenicol modifying enzymes and showed resistance to chloramphenicol (MIC 32 to > 128 mg/L) (Fig. 3 and Table S1). The most common resistance determinant was a gene (*catA13*) coding for a type A-13 chloramphenicol O-acetyltransferase (WP_040564913.1; Fig. 4). This resistance gene was present in all except five chloramphenicol resistant isolates, either alone or in combination with *catA9* (*n* = 14), encoding a type A-9 chloramphenicol O-acetyltransferase (WP_001010387.1). The *catA9* determinant was also present in the residual five chloramphenicol resistant isolates. The point mutation in *catA9* observed in two chloramphenicol resistant *C. coli* isolates (BfR-CA-16261, BfR-CA-18728), leading to a single amino acid substitution (p. A197T) in CatA9, was falsely annotated as *catTC* gene with an internal stop codon by AMRFinderPlus.

One sensitive *C. coli* (BfR-CA-16259) displayed a *catA* gene, which corresponded to a protein of 262 amino acids and was N-terminally identical to CatA13 until P179 (CatA13_p.L180-K207delins180-262). The C-terminus was different from CatA proteins. The gene was “correctly” found as a partial *catA13* gene by AMRFinderPlus.

Furthermore, 15.2% *C. coli* (*n* = 15) and 1.9% *C. jejuni* (*n* = 3) were highly resistant to florfenicol (MIC values of > 16 mg/L; Table S1). The two resistance genes coding for a **f**lorfenicol **ex**porter protein **A** (*fexA*) and an ABC-F type ribosomal protection protein (*optrA*), respectively, were found to be associated with high level florfenicol resistance. In the majority of highly resistant isolates, both genes were present (n_*C.coli*_=12, n_*C.jejuni*_=3); just three *C. coli* isolates either harbored *fexA* (BfR-CA-15989) or *optrA* (BfR-CA-16261, BfR-CA-18728), indicating that either gene might be sufficient for high level florfenicol resistance. Medium-level resistance (MIC 8–16 mg/L) could not be attributed to the presence of a genetic determinant (*n* = 11; Table 3 and S1).

### Resistance to β-Lactams

Genes encoding oxacillinases (class D β-lactamases) of the OXA-61- or -184-like family were identified in 215 (89.6%) and 244 (96.1%) isolates from Germany and Vietnam (Table S1 and S3) by AMRFinderPlus, respectively. The predominant variant found was *bla*_OXA−193_, which accounted for 61.2% of identified *bla*_OXA_ genes (281/459). Other variants that were found more frequently were *bla*_OXA−489_, *bla*_OXA−184_, and *bla*_OXA−460_. Overall, 21 different *bla*_OXA_ genes were identified and further variants with yet unknown point mutations, belonging to either *bla*_OXA−61_ or *bla*_OXA−184_ family genes. Genes of the OXA-184-like family were only detected in *C. jejuni* isolates. Susceptibility to ampicillin was tested in approximately 30% of the isolates, demonstrating resistance to ampicillin with MIC values ranging from 32 to > 512 mg/L in the presence of a *bla*_OXA_ gene, except for one strain. This *C. jejuni* from Germany (BfR-CA-14940) displayed a MIC of 16 mg/L ampicillin, just below the ECOFF for resistance, but carried a *bla*_OXA−193_ gene. We analyzed the promoter of the *bla*_OXA_ gene in this isolate using the Geneious software. It was found previously that a transversion (G to T) at position − 57 restored the Pribnow box, leading to up-regulation of *bla*_OXA_ and high-level ß-lactam resistance [69]. Indeed, this point mutation was missing in BfR-CA-14940, thus potentially explaining the low observed MIC for ampicillin. Consistently, isolates carrying *bla*_OXA_ with lower MIC values between 32 and 64 mg/L also did not harbor the optimal Pribnow box for increased *bla*_OXA_ transcription. There was one exception to the rule (BfR-CA-16023), carrying a *bla*_OXA_ gene with the non-optimal Pribnow box but displaying a MIC value of 256 mg/L. Furthermore, one *C. coli*_*VN*_ isolate was detected, which did not harbor a *bla*_OXA_ gene, but showed slight ampicillin resistance just above the ECOFF (MIC = 32 mg/L).

### Resistance to Nourseothricin

The resistance determinant *sat4*, encoding a streptothricin N-acetyltransferase, accounted for resistance to nourseothricin, a mixture of streptothricins C, D, E and F. Isolates carrying *sat4* showed MIC values between 8 and 512 mg/L nourseothricin, while the respective sensitive isolates without *sat4* had MICs of ≤ 1–2 mg/L. Although an ECOFF value is not yet officially published, we defined > 4 mg/L as elevated non-wildtype MICs for our study to categorize sensitive and resistant isolates (Table 1). The *sat4* resistance determinant was more common in Germany with 21.7% *C. coli* (*n* = 25/115) and 20.8% *C. jejuni* (*n* = 26/125) harboring *sat4* compared to only five isolates from Vietnam. The translated protein sequences showed high similarity to the reference protein WP_000627290.1.

### Prediction of localization and mobilization of AMR genes

The tool Platon v1.6 was used for annotation of plasmid localization of AMR genes based on short-read data. For verification, selected isolates were also processed by Oxford Nanopore long-read technology (n = 14). All fourteen genomes could be closed using the Unicycler hybrid assembler and the chromosomes displayed a size between 1.62 and 1.82 Mb, while six isolates carried an additional circular plasmid of 3.3 kb to 52 kb (Table 4). AMR genes in these isolates were mostly found on the chromosome. Only two plasmids carried either a *tet*(O) gene (BfR-CA-15687) or an operon containing *tet*(O/32/O) *– aadE*2*_*Δ1-415 *– sat4 – aph(3’)-IIIa* (BfR-CA-16737). We asked whether the current plasmid prediction from short-read data using the Platon tool corresponded to the closed genomes/plasmids upon long-read sequencing within our dataset. We observed that the annotation of “plasmid contigs” by Platon overestimated plasmid existence in three isolates (false positives), while missing the plasmid in one isolate (false negative, Table 4). The prediction of the presence of either/and (i) a circular plasmid, (ii) mobilization or (iii) conjugation elements (Table S1, column BC, BD, BE) led to missing two plasmid-containing isolates. However, false positive results were lacking. If this conservative filter was applied for all short-read data (Table S1, column BF > 0), 183 of the total 494 isolates were predicted to carry one or multiple plasmid/s. However, prediction of plasmid-location of AMR genes seemed to be inacurate based on the Platon tool optimized for other bacteria such as *Escherichia coli*: only one out of the two plasmids, which contained AMR gene/s, was detected by Platon and a further four isolates were falsely annotated as carrying AMR genes on plasmids based on short-read data.

**Fig. 5:**
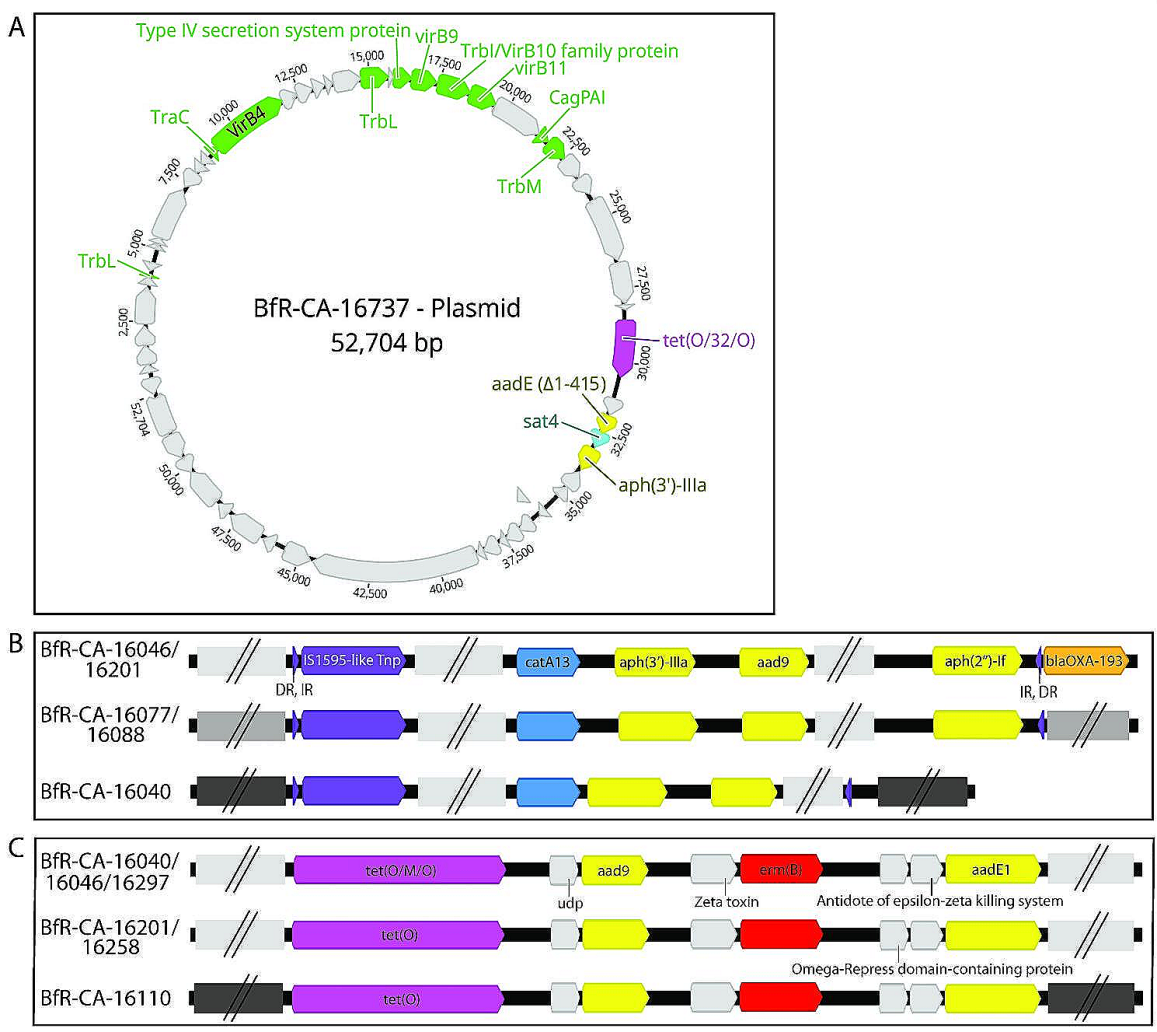
Mobilizable MDRI clusters and AMR genes in *Campylobacter* spp. identified by long-read sequencing. Localization of AMR clusters on **A**, plasmid, **B** and **C**, the chromosome, with **B** in proximity to transposase genes. All elements are mobilizable by natural transformation in *Campylobacter* spp. In addition, conjugative transfer (**A**) and transposition (**B**) is likely to occur. In **A**, genes associated with type IV secretion/conjugation are depicted in green; in **B**, transposase genes and associated direct (DR) and inverted repeats (IR) are marked in purple. AMR genes from different antimicrobial classes are depicted in different colors; blue, *catA* genes, yellow, aminoglycoside resistance genes; orange, *bla*_OXA_ gene; light purple, *tet*(O) variant genes; red, *erm*(B) genes. Grey arrows, non-AMR related genes; grey boxes with two vertical lines indicate clusters of non-AMR related genes, with homology indicated using identical shading

Based on long-read sequencing data and hybrid assemblies using Unicycler, we further investigated the localization of AMR gene clusters and their mobilization potential using AMRFinderPlus. In principle, we found three types of AMR gene localizations that suggest different mobilization of AMR genes (Fig. 5). As mentioned above, plasmid localization of AMR genes was rare. Only one *tet*(O) gene in a *C. coli* isolate and the operon structure *tet*(O/32/O) *– aadE*2*_*Δ1-415 *– sat4– aph(3’)-IIIa* in a *C. jejuni* were plasmid-located within the long-read sequenced isolates (Fig. 5A), thus, being transferable via conjugation. Chromosomal AMR genes, like e. g. the gene cluster *tet*(O) *– aad9 – erm*(B) *– aadE*1 can be transferred by natural transformation, depending on homologous recombination. Likewise, we observed that the gene context of this MDRI was rather stable in the analyzed long-read sequenced isolates, with five out of six isolates displaying homologous gene context flanking the MDRI (Fig. 5C). Also the chromosomal *aac(6’) -Ie/aph(2’‘) -Ia– aadE*1 *– tet*(O)x_ΔC−terminus_ gene cluster was embedded in a highly conserved genomic region in the analyzed four isolates, which is expected for mobilization via natural transformation. However, we frequently found chromosomal MDRI in proximity to a transposase gene. For example the *catA13 – aph(3’) -IIIa – aad9* MDRI was situated in three different chromosomal contexts with and without additional adjacent AMR genes in five analyzed isolates (Fig. 5B). Hence, this MDRI was putatively disseminated by natural transformation and transposition, thereby enhancing the movement within a bacterial chromosome but also among the bacterial population. A similar mechanism of transfer might be predicted for other MDRIs as well as for single AMR genes in proximity to transposase genes, e. g. *aph(2’’) -If– aph(3’) -IIIa* in *C. coli* BfR-CA-16297, *aadE*3 *– sat4– aph(3’) -IIIa* in *C. jejuni* BfR-CA-19301, *lnu*(C) or a second copy of *aph(3’) -IIIa* in *C. jejuni* BfR-CA-16077 and BfR-CA-16088, *tet*(O/M/O) *– catA9 – fexA – optrA – tet*(L) in *C. coli* BfR-CA-15991 and *tet*(O/32/O) -*aph(2’’) -li*_*1*_* – aph(3’) -IIIa – aad9 – aadE*1 *– tet*(O)_ΔN−terminus_ in *C. coli* BfR-CA-19087 (Figure S8).

## Discussion

The study aimed to improve AMR diagnostics of thermotolerant *Campylobacter* spp. by elucidating the reliability of predictions for antimicrobial resistances from whole genome sequence data. Within nearly 500 investigated isolates, whole genome cgMLST results suggested a broad diversity of isolates, constituting a suitable data source for in-depth AMR analysis. We detected 14 different resistance genes and genes with point mutations in isolates from Germany and 22 different AMR determinants associated with antibiotic resistance in the *Campylobacter* spp. population from Vietnam. Each identified resistant determinant was correlated to phenotypic resistance against the respective antimicrobial. Any discrepancies were re-analyzed. Our study showed high rates of aminoglycoside, (fluoro-)quinolone, macrolide, phenicol and tetracycline resistance in isolates from Vietnam, which is likely related to the extensive use of antibiotics on farms [24] and comparable to those previously reported in other Asian countries such as China, Malaysia, and the Philippines [69–72]. Resistance to (fluoro-)quinolones and tetracycline was also frequent in isolates from Germany, while resistances to aminoglycosides and macrolides were comparably low, which is in line with recent data from Germany [73].

Our main question was whether current next generation sequence data analysis pipelines are prepared for appropriate detection of potential worldwide spread of multi-resistant *Campylobacter* spp. With our systematic approach we observed five principle discrepancies between pheno- and genotype in thermotolerant *Campylobacter* spp.

### Missing or falsely annotated AMR genes in databases

First, certain AMR genes were either missing in the AMRFinderPlus and ResFinder databases (*aadE*3), although previously published [74] or falsely annotated to confer resistance in *Campylobacter* spp. in the AMRFinderPlus database (ribosomal L22 protein A103V mutation). Despite the previous suggestion that the point mutation A103V in the ribosomal protein L22 may confer resistance to macrolides [66], our findings do not show a correlation between this mutation and erythromycin resistance. This is consistent with the conclusion reached by others, who also found no association between A103V and resistance to macrolides [30, 75]. Furthermore, the mosaic gene variant *tet*(O/32/O) is also missing in the AMRFinderPlus database (version 2023-08-08.2) and was identified by the pipeline as *tet*(O) with reduced identity (~ 93%), thus, at least not causing a pheno-/genotype discrepancy. However, ResFinder database 2.1.0 includes this variant. The above mentioned inconsistencies can be easily addressed by curation and harmonization of the databases.

### Detection of *tet*(O), *aadE* genes and *aad9* partially failed due to frequently observed presence of multiple copies or variant genes

Second, short-read sequencing eventually failed or falsely detected partial (inactive) AMR genes, if multiple copies and/or homologous mosaic genes were present. This was, in particular, the case for *tet*(O) but also for *aadE* gene variants and multiple copies of *aad9*. In the *Campylobacter* population from Vietnam, there was a high prevalence of isolates with either two identical or two distinct variants of the (mosaic) tetracycline resistance genes (Table S1). The assembler used may have encountered difficulties in generating complete resistance gene sequences from raw reads due to regions of ambiguity within the assembly process. Consequently, either incomplete genes were identified in these isolates, or the sequencing reads were inadequate in length and did not meet the pipeline’s coverage threshold, which resulted in “absence” of AMR gene detection (Table 3). As proof of principle SKESA as alternative assembler was used for the assembly of short-read sequencing data of 10 isolates, for which detection of some AMR genes failed using the shovill assembler (Table S2). However, the results were similar, except that in one isolate the full-length copy of *aad*9 was, in addition, falsely detected as “partial” upon SKESA assembly. In another isolate, in which *tet*(O) was missing upon shovill assembly, SKESA assembly led to the detection of a partial *tet*(O). In a study with commensal *E. coli*, short-read sequencing was capable of detecting only one copy of each duplicated resistance gene, yet the authors did not observe partial or unidentified genes arising from allelic variants [76].

Under our test conditions, we did not observe any functional differences between the *tet*(O) or *aadE* variants, nor enhanced resistance levels were detected, if isolates carried multiple copies of resistant gene variants (verified by long-read sequencing). Thus, so far the impact or purpose for redundant genetic determinants in *Campylobacter* spp. remains unknown. It might be speculated that redundant genes are located in a gene context with essential/other AMR genes, thereby, being co-transferred. Most initial discrepancies from AMRFinder with its default thresholds were resolved by manual search of missing genes via ABRicate and by mapping of raw reads to reference genes using Geneious Prime (as exampled in Figure S4). Mosaic tetracycline genes such as *tet*(O/M/O) and *tet*(O/32/O) variants were previously found [77, 78] and in this study, we showed differential distribution of these variant genes in different *Campylobacter* populations. Yet, the complexities arising from the diverse recombinant forms of *tet*(O) within *Campylobacter* isolates from Vietnam could not be conclusively resolved unless long-read sequencing was applied. Long-read sequencing by Oxford Nanopore Technology deciphered multiple copies of AMR genes (including multiple identical genes and or partial genes) and, furthermore, revealed AMR gene localization (Table 4), which was frequently inconsistent with predictions from short-read sequencing. Hence, a combination of short- and long-read sequencing may circumvent inconsistencies caused by the presence of multiple AMR gene (variants) with the additional benefit of identification of AMR gene location.

### Novel point mutations in *tet*(W) led to AMR gene inactivation, while *aad9* was identified as phase variable gene

Third, while some partial genes harboring point mutations were correctly identified by the pipeline, we identified novel point mutations D171N/G579D in Tet(W), leading to a tetracycline sensitive phenotype (BfR-CA-16942 and BfR-CA-18353). Furthermore, in case of the *aad9* gene, around 70% of the isolates were annotated to display a truncated inactive version of *aad9*, but those isolates were indeed resistant to spectinomycin. Next to assembly problems due to multiple copies of *aad9*, we revealed weakness of the assembling process to correctly identify the poly-C tract variant of functional *aad9*. This was probably due to a mixture and ambiguity of raw reads with different number of cytosines within this novel phase variable gene (Figure S7). As proof of principle we reselected an isolate annotated as harboring an inactive *aad9* gene on spectinomycin and after re-sequencing, we were able to correctly identify the full-length *aad9* gene. This observation is in agreement with reversion to a functional gene by insertion/deletion of cytosines, explaining the phenotypic resistance observed in the antimicrobial sensitivity tests. Thus, we concluded that *aad9* is frequently inactivated by frameshifting, but the isolates keep resistance to spectinomycin as a bacterial population due to the reversion of the frameshift. Phase variation was proposed an important mechanism for regulation of several genes in *Campylobacter* spp., in particular for host response, like e.g. the *flgR/S* system, essential for motility [79, 80]. Here, it might balance the cost for AMR gene carriage and suggests prolongation of persistence of the AMR gene.

### MIC values just above the cut-off probably display non-specific resistance due to enhanced efflux and/or decreased inward diffusion

Fourth, discrepancies were identified for isolates with MIC values close to the cut-off value. Most frequently, we found isolates without any known resistance determinant but with slight resistance according to the current ECOFF or elevated non-wildtype MICs. This was the case for four lincosamide and eleven florfenicol resistant isolates and for one isolate resistant to ampicillin (Table 3). Low level resistance without known gene determinants might be promoted by increased efflux or decreased influx mechanisms [80–83]. It has been previously reported that inactivation of the ABC-efflux transporter CmeABC led to increased sensitivity to a variety of antimicrobials such as (fluoro-)quinolones, macrolides, phenicols and tetracyclines [84, 85]. Low level ampicillin resistance was due to the presence of the non-optimal Pribnow box in the promotor region, if *bla*_OXA_ genes were present (see above and [69, 86]). We identified a further exceptional isolate (BfR-CA-16023) carrying a *bla*_OXA_ gene with the non-optimal Pribnow box but displaying a MIC value of 256 mg/L. As for the slightly ampicillin resistant isolate BfR-CA-19104 without *bla*_OXA_, there might be additional efflux and/or decreased influx mechanisms, which await further investigations.

### Unknown resistance mechanisms in *Campylobacter* spp. remain elusive

Fifth, we found isolates harboring unknown resistance mechanisms. One gentamicin resistant isolate from German turkey cecum (BfR-CA-15687) did not harbor any of the known resistance determinants but repeatedly showed high level resistance to gentamicin (MIC > 16 mg/L). The isolate was also resistant to (fluoro-)quinolones (GyrAT86I) and tetracycline (*tet*(O)) and carried a *bla*_OXA−489_ gene. Further studies are needed to identify the unknown gentamicin resistance mechanism.

As previously confirmed by other studies and reiterated by our WGS results, the single point mutation T86I in the “quinolone resistance determining region” (QRDR) of the gyrase subunit A confers resistance to (fluoro-)quinolones in *Campylobacter* spp. [33, 87, 88]. This widespread resistance in *Campylobacter* isolates is likely due to the demonstrated fitness advantage that it confers at least in some *C. jejuni* isolates [89]. However, we found several isolates, harboring the point mutation GyrA T86I and displaying high-level resistance to ciprofloxacin but complete sensitivity to nalidixic acid (Table 3 and S1). This phenomenon was previously found by others [29, 90] but the underlying mechanism is yet unsolved. We conclude that the point mutation alone is not sufficient for both resistances to ciprofloxacin and nalidixic acid and the overall (fluoro-)quinolone resistance mechanism in *Campylobacter* remains enigmatic.

### Fitness costs of AMR and Impact of AMR gene localization on transfer and spread

ONT sequencing and hybrid assembly of long- and short- reads led to closure of circular contigs, the chromosome and potential plasmids. Hybrid assembly data resulted in improved identification of multiple AMR gene(s) variants, which were non-resolvable by only short-read analysis. Interestingly, *Campylobacter* populations in Germany and Vietnam showed distinct patterns of gene variants, e.g. *tet*(O/M/O) in Vietnam and *tet*(O/32/O) in Germany. The reason for the acquisition of redundant resistance mechanisms by the isolates is uncertain. In our analysis we could not find enhanced levels of resistance due to multiple resistance determinants, since the presence of one copy already led to high level resistance of the AMR investigated. However, if selection is exerted on AMR genes situated within AMR gene clusters, also neighboring AMR genes are co-selected and transferred from one isolate to another. We conclude that AMR genes in *C. jejuni* and *C. coli* were frequently organized in mobilizable MDRIs next to transposase genes and different MDRIs harbored multiple AMR genes with analogous function. Hence, these isolates appear to be perfectly prepared for a changing selective environment and additionally harbored transiently non-functional AMR genes, which might be restored under selection pressure.

Interestingly, most AMR genes appeared to be chromosomally located, frequently in association with transposase genes (Fig. 5). Plasmid prediction from short-read data was limited, while long-read data identified 43% strains carrying a plasmid (n = 6/14). From these isolates, only two plasmids were identified with AMR genes, one harbored *tet*(O), the other *tet*(O/32/O) *– aadE*2_Δ1-415 *– sat4– aph(3’)-IIIa*. This is consistent with previous findings in the literature, where plasmids containing tetracycline resistance genes were reported in *Campylobacter*, such as the self-transferable plasmid pTet and *tet*(O) associated AMR gene clusters [91, 92]. Previous research has demonstrated the existence of the resistance gene cluster *aadE – sat4 – aph(3’) -IIIa*, located on both the chromosome and plasmids, in *C. jejuni* and *C. coli* isolates [74, 92–95]. These findings align with our results from long-read sequencing. It is noteworthy that the use of streptothricin was restricted to the former German Democratic Republic, and ceased by 1989 at the latest, while therapeutic use in humans has been halted due to its nephrotoxicity [96]. It is possible that the *sat4* gene is conserved to some degree as it is co-flanked within the aminoglycoside resistance conferring genes *aadE* and *aph(3’)-IIIa*, that might provide an advantage to *Campylobacter* in Germany and explain the observed preferential presence of *sat4* in isolates from Germany.

Spread of macrolide resistance is of great concern, since in particular macrolides are the drug of choice to treat campylobacteriosis in humans [9]. The point high level resistance conferring mutation A2075G in the 23S rRNA was shown to result in a substantial decrease in bacterial fitness among *C. jejuni* [97, 98]. This fact may explain its low prevalence in areas with a comparably low selection pressure. In regions with high selection pressure, such as Vietnam [25, 99] this mutation was more frequently found (Fig. 3). Additionally, high-level resistance to macrolides and/or lincosamides is also conferred by the emerging resistance gene *erm*(B), which was first described in a *C. coli* strain isolated from swine in China [100]. We showed in our study that phenotypic resistance testing with erythromycin cannot distinguish the presence of the 23S rRNA point mutation from that of *erm*(B), since the MIC distribution of both resistant determinants was comparable (Figure S3). As also observed in our study (Fig. 5), *erm*(B) has already been shown to be part of different MDRIs [98, 101, 102] and probably derived from Gram-positive bacteria [103]. In the ONT-analyzed isolates, *erm*(B) was located on the chromosome with a rather conserved gene context, suggesting mobilization via natural transformation (Fig. 5). We previously showed that natural transformation in *C. jejuni* was a highly efficient process and occurred most frequently under microaerobic conditions at neutral pH, found in the natural hosts [104].

The *catA13– aph(3’)-IIIa– aad9* cluster was one of the AMR clusters found in proximity to transposase genes (Fig. 5; Table 4). As expected for transposable elements, the AMR cluster context was rather diverse, with occasional acquisition of additional nearby located AMR genes, like *aph(2’’) -If* and *bla*_OXA−193_. Interestingly, in another study from China the two resistance genes *fexA* and *optrA* were found together as part of an MDRI, which aligns with the data we collected [105]. Given that the two genes were also identified in close proximity to transposases within operon structures among isolates from Vietnam, it is highly probable that they will continue to disseminate. Although chloramphenicol is not commonly used in human medicine due to its bone marrow toxicity, it is still reserved for the treatment of severe infections such as certain types of meningitis, rickettsiae, or typhoid fever [105–109].

The high prevalence and frequent redundant presence of multiple homologous and analogous resistance genes, e. g. *aph(2’’)-If*, *aac(6’)-Ie/aph(2’’)-Ia*, *aph(3’)-IIIa* and *aadE* in particular, in the isolates from Vietnam, may reflect regular selection of MDRI, resulting in AMR accumulation. In general, high resistance to aminoglycosides should be regarded as concerning as they are considered a high-priority critically important antimicrobial class according to the World Health Organization [110].

## Conclusion

Our results highlight the extensive presence of various AMR genes and gene variants, as well as point mutations associated with AMR in the investigated *Campylobacter* population. The approach corroborated the necessity for continuous update of databases with respect to novel AMR gene (variants), point mutations leading to (transient) inactivation of AMR and for including long-read sequencing for improved detection of redundant AMR genes and AMR gene locations. Limitations of gene detection from short-read assemblies can partially be dealt with by lowering required coverage thresholds and complementing analysis with read mapping approaches. Furthermore, yet unknown mechanisms for gentamicin and (fluoro-)quinolone resistance, transiently inactive AMR genes and mobilization of MDRI await further investigation. The findings showed elevated levels of resistance depending on the origin of isolation, emphasizing the need for improved surveillance and diagnostics of AMR in thermotolerant *Campylobacter* spp. along the food production chain globally.

### Electronic supplementary material

Below is the link to the electronic supplementary material.

Supplementary Material 1

Supplementary Material 2

## Data Availability

Raw read sequences and either complete annotated genomes or draft genomes were published within the BioProjects No. PRJNA562653, PRJNA595957, PRJNA648048 and PRJNA872862. All data supporting the findings of this study are provided within the paper and its supplementary information. All additional information are available upon request from the authors.

## Abbreviations

AMR: Antimicrobial Resistance
CAMHB/FCS: Cation-supplemented Mueller-Hinton broth with 5% fetal calf serum
CC: Clonal Complex
CI: Confidence Interval
ColbA: Columbian Blood Agar
ECOFF: Epidemiological Cut Off
EDTA: Ethylenediaminetetraacetic Acid
FCS: Fetal Calf Serum
mCCDA: Modified Charcoal-Cefoperazone Deoxycholate Agar
MDRI: Multidrug Resistance Island
MIC: Minimum Inhibitory Concentration
MLST: Multi-locus sequence typing
MST: Minimum Spanning Tree
ONT: Oxford Nanopore Technology
OR: Odds Ratio
QRDR: Quinolone resistance determining region
SRA: Sequence Read Archive
WGS: Whole-Genome Sequencing
WHO: World Health Organization
