# Supplementary material for: Identification of knowledge gaps in whole-genome sequence analysis of multi-resistant thermotolerant Campylobacter spp
Source: BMC Genomics. 2024 Feb 8;25:156. doi: 10.1186/s12864-024-10014-w (PMC10851486; doi:10.1186/s12864-024-10014-w)
Supplement: Supplementary file 2 — Supplementary Material 2 [file 12864_2024_10014_MOESM2_ESM.pdf]

Supplementary Figures

Identification of knowledge gaps in whole-genome sequence analysis of multi-resistant thermotolerant *Campylobacter* spp.

Michael Zarske, Huong Quynh Luu, Carlus Deneke, Marie-Theres Knüver, Maja Thieck, Ha Thi Thu Hoang, Nancy Bretschneider, Ngoc Thi Pham, Ingrid Huber, Kerstin Stingl

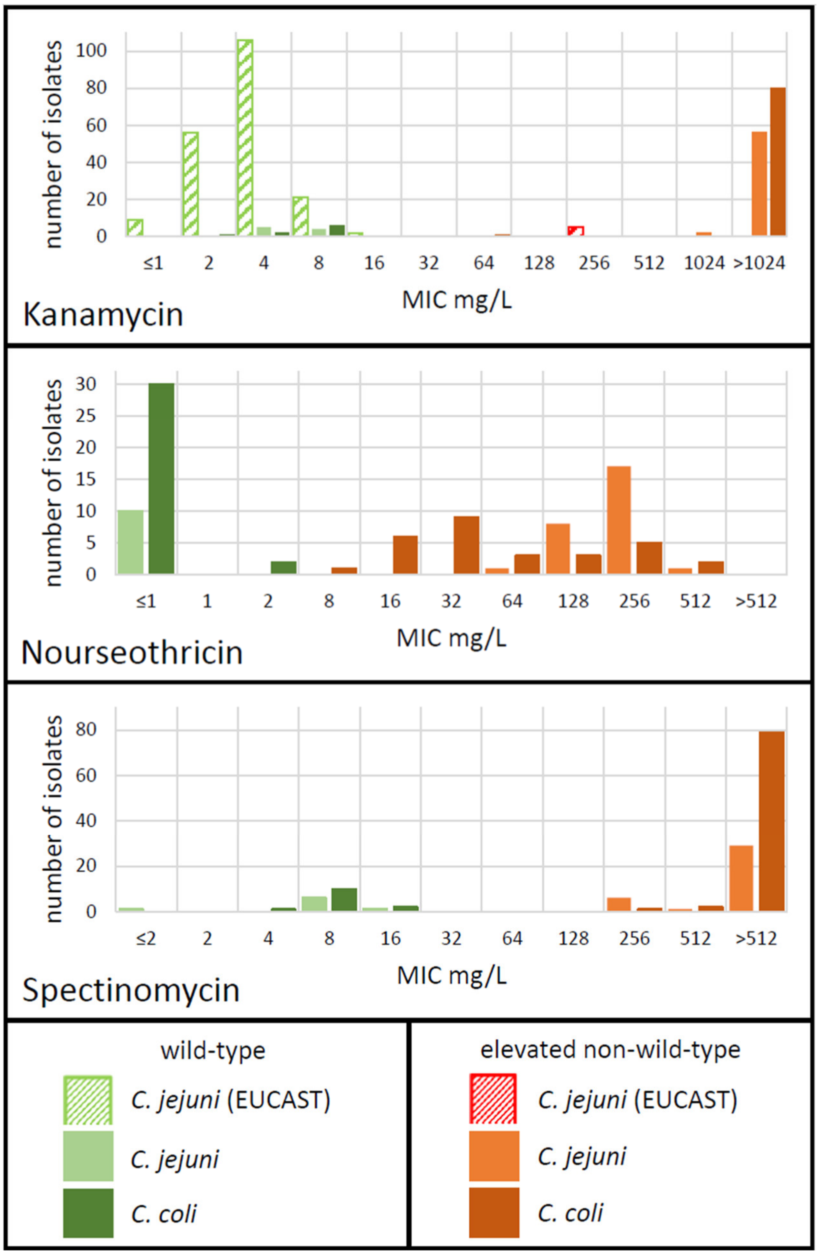

Figure S1. Distributions of MIC values of *Campylobacter* spp. isolates for antimicrobials lacking an epidemiological cutoff (ECOFF) value. Isolates were categorized into wild-type (green) and elevated non-wild-type (red) based on their MICs. Hatched bars, data from EUCAST (last accession on 11/16/2023); filled bars, results of this study.

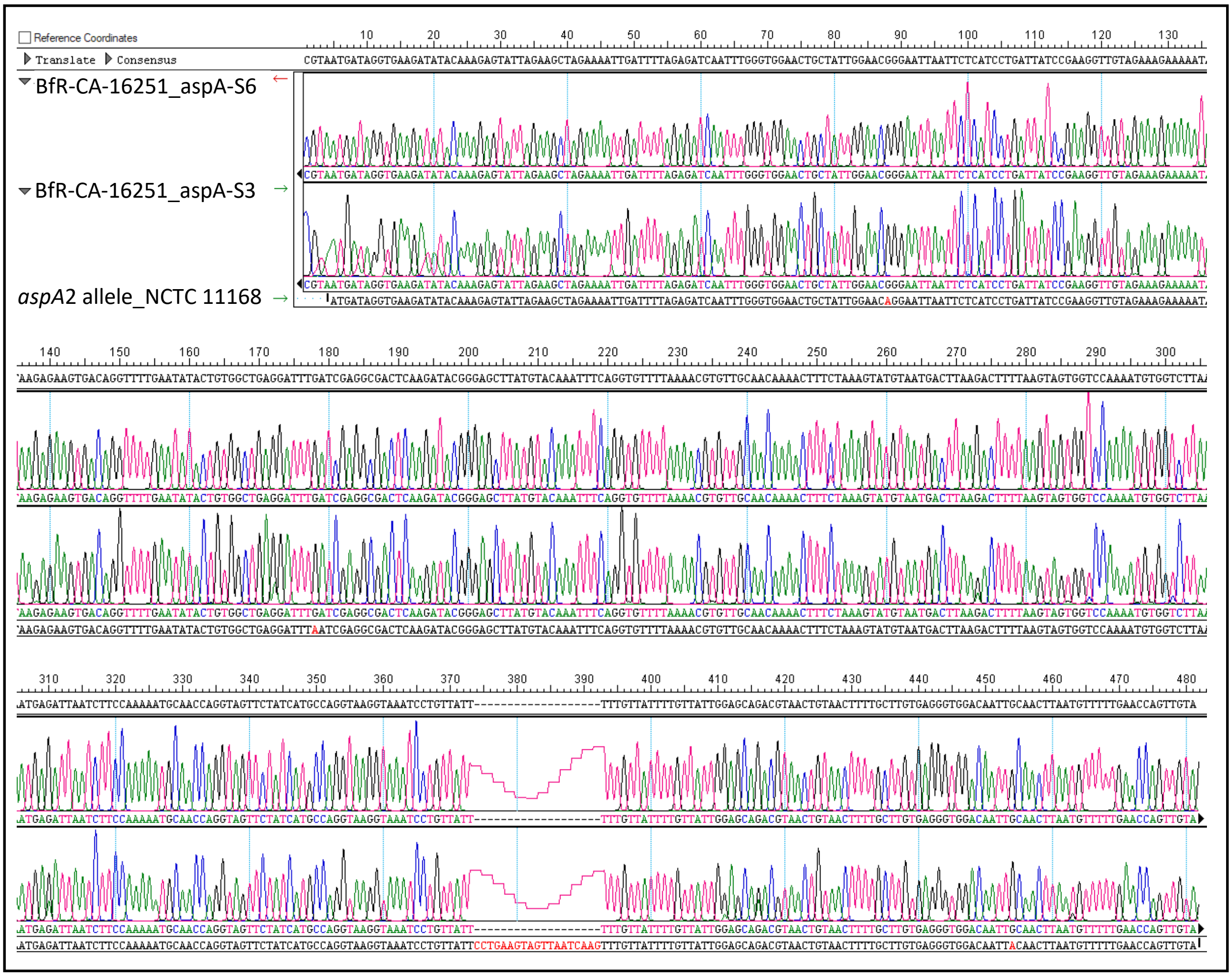

Figure S2. Sanger sequencing traces confirmed a deletion of 19 bp in *aspA* of BfR-CA-16251. A PCR fragment of *aspA*, amplified with primers aspA-A9/-A10 from BfR-CA-16251, was subjected to Sanger sequencing using primers aspA-S3 and aspA-S6. The obtained sequences were aligned to the MLST allele 2 of *aspA*, as present in *C. jejuni* NCTC 11168 (NC_002163.1), utilizing Lasergene SeqMan Pro (Lasergene 17, DNASTAR Inc., Madison, WI, USA). The deletion of 19 bases (highlighted in red) within *aspA* in BfR-CA-16251, also observed upon assembly of short-read NGS data, was confirmed. The ruler annotates the base positions in the *aspA* fragment, defined as an allele for MLST at PubMLST (45).

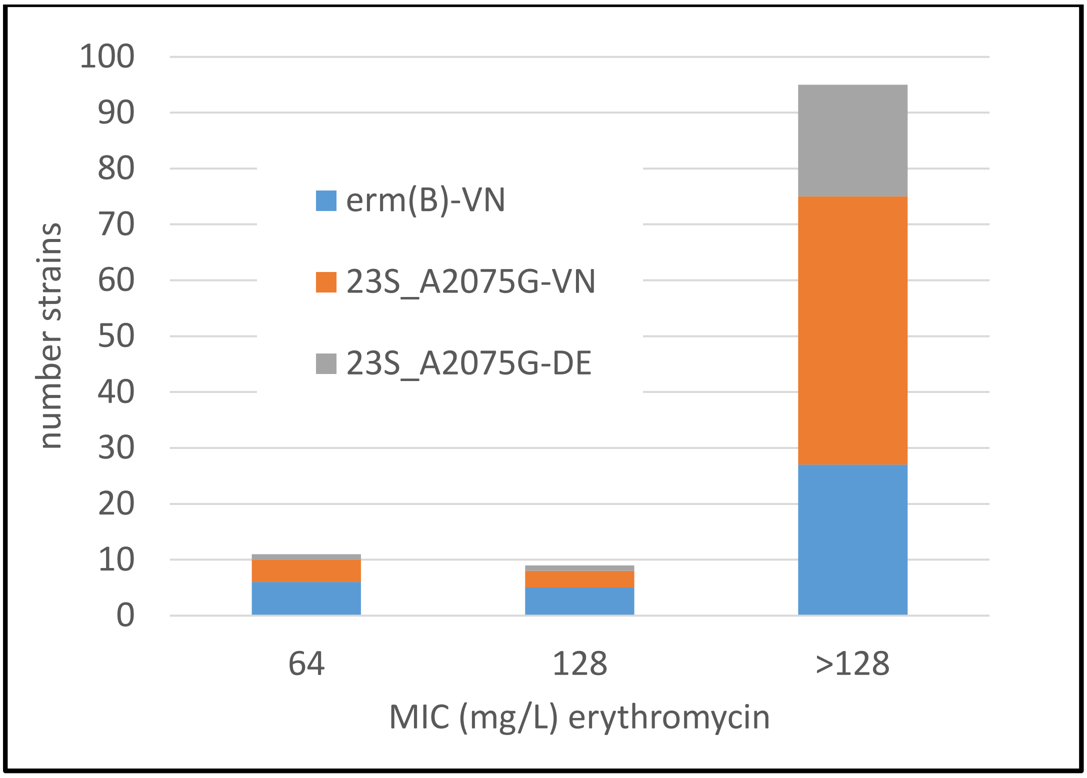

Figure S3. Minimum inhibitory concentration of erythromycin is similarly distributed among *erm*(B) and 23S_A2075G carrying *Campylobacter* isolates. The obtained results demonstrate that MIC values of erythromycin cannot differentiate between the presence of *erm*(B) and 23S_A2075G.

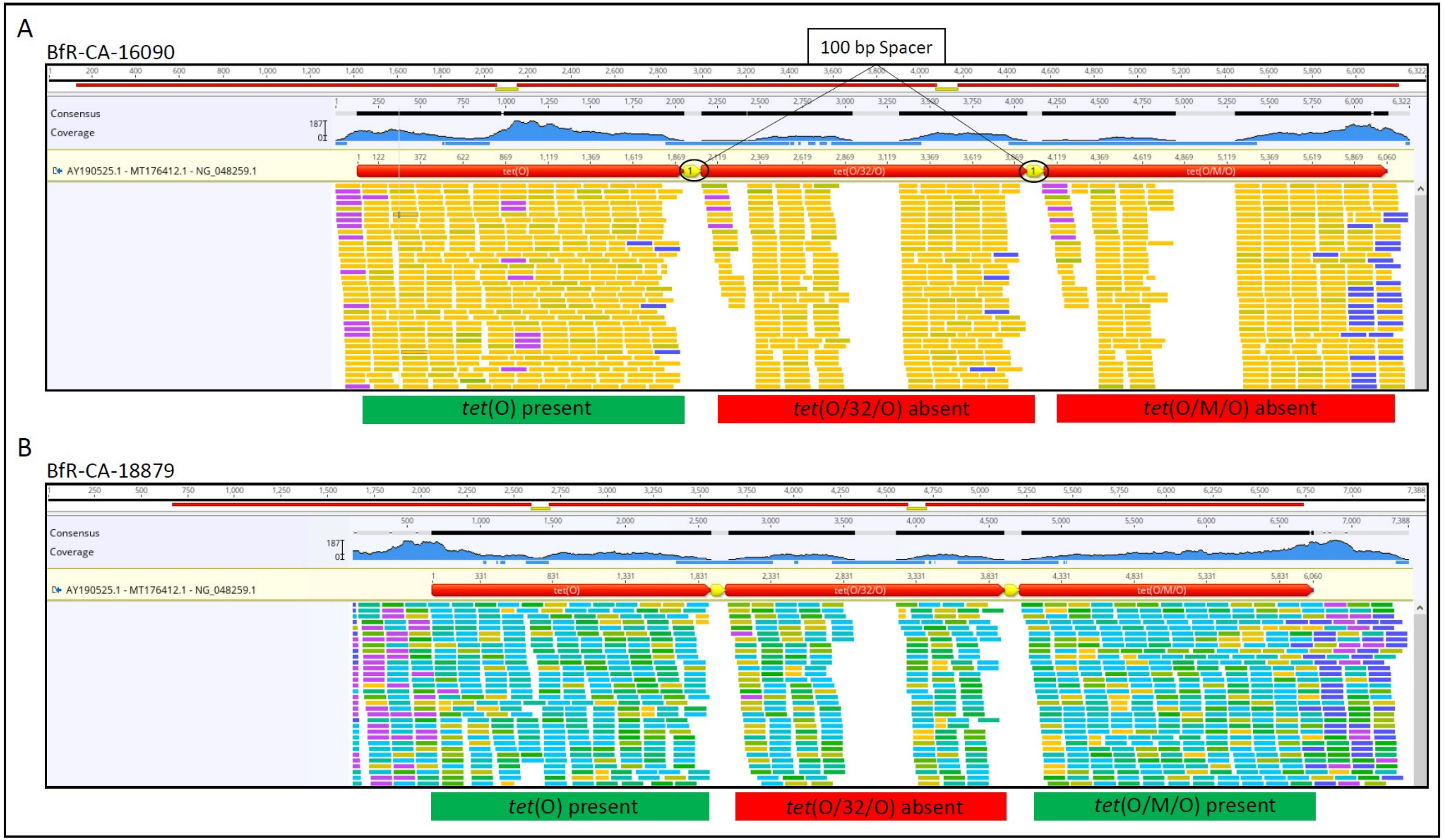

Figure S4. Identification of multiple copies of *tet*(O) variant genes using Geneious Prime. An artificial template of three reference genes, *tet*(O) (Acc. AY190525.1), *tet*(O/32/O) (Acc. MT176412.1) and *tet*(O/M/O) (Acc. NG_048259.1), separated by 100 bp spacers (highlighted in yellow) was created for mapping of trimmed raw reads from isolates showing tetracycline resistance but displaying absence or only partial *tet*(O) genes according to AMRFinderPlus. Two examples of strains carrying multiple *tet*(O) variants are shown. **A.** Read mapping of BfR-CA-16090 to the template indicated full-length presence of *tet*(O). **B.** Mapping reads of BfR-CA-18879 revealed presence of both *tet*(O) and *tet*(O/M/O) genes.

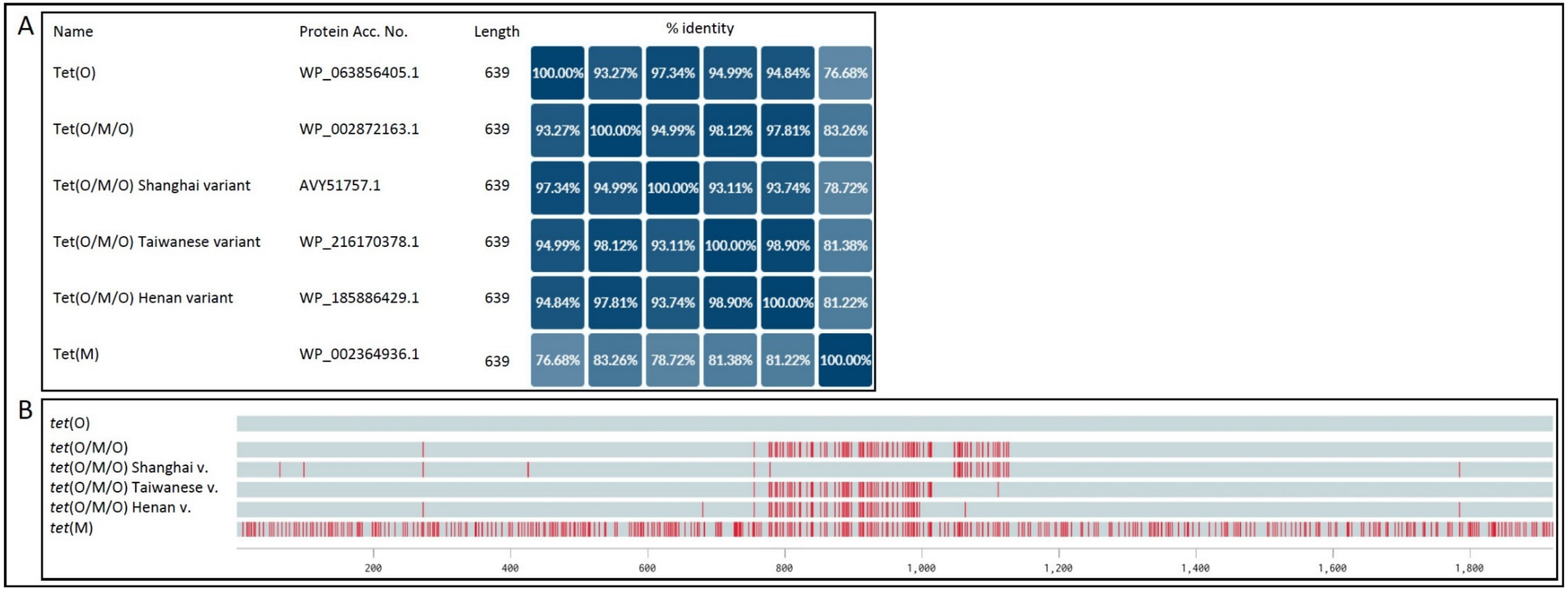

Figure S5. Visualization of different *tet*(O/M/O) variants found in Vietnamese *Campylobacter* spp.. **A** percent of amino acid identity shared among the reference sequences. NCBI Protein Acc. No. and respective lengths are illustrated. Percent identity matrix generated with UniProt Align tool (56). **B** Nucleotide Alignment highlighting the areas of *tet*(M) introgression into *tet*(O) among the different *tet*(O/M/O) variants found in Vietnamese isolates. NCBI Nucleotide Accession of genes shown are presented in Table S3. Alignment generated with Benchling's DNA sequence alignment tool (110).

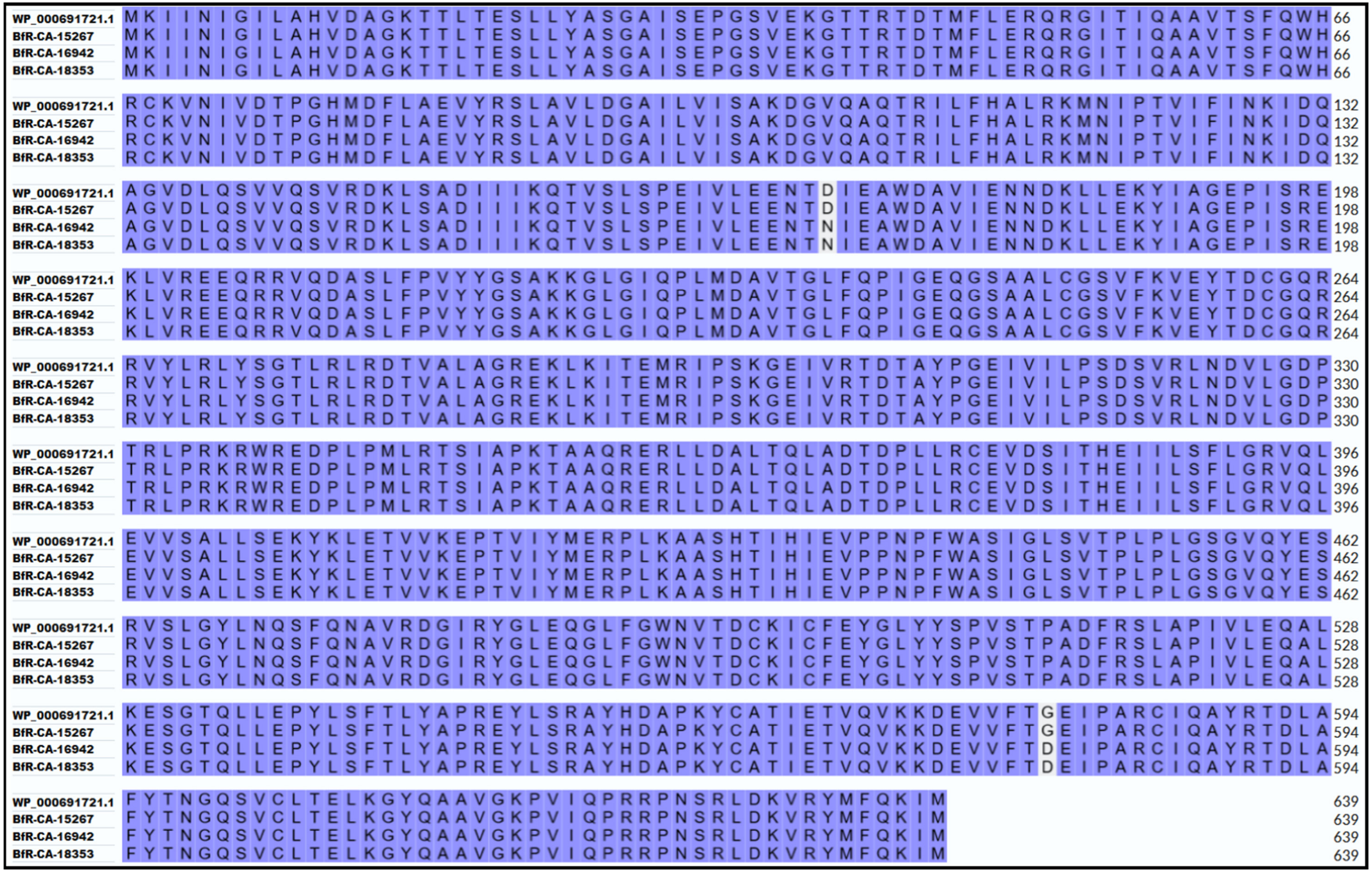

Figure S6. Protein Alignment of reference Tet(W) (WP_000691721.1) and translated gene assemblies of the three isolates harboring *tet*(W). The tetracycline sensitive isolates, BfR-CA-16942 and BfR-CA-18353, showed two amino acid substitutions (D171N and G579D) in Tet(W), while Tet(W) of the tetracycline resistant BfR-CA-15267 was 100 % identical to the reference protein. Protein alignment was created with Uniprot Align tool (56).

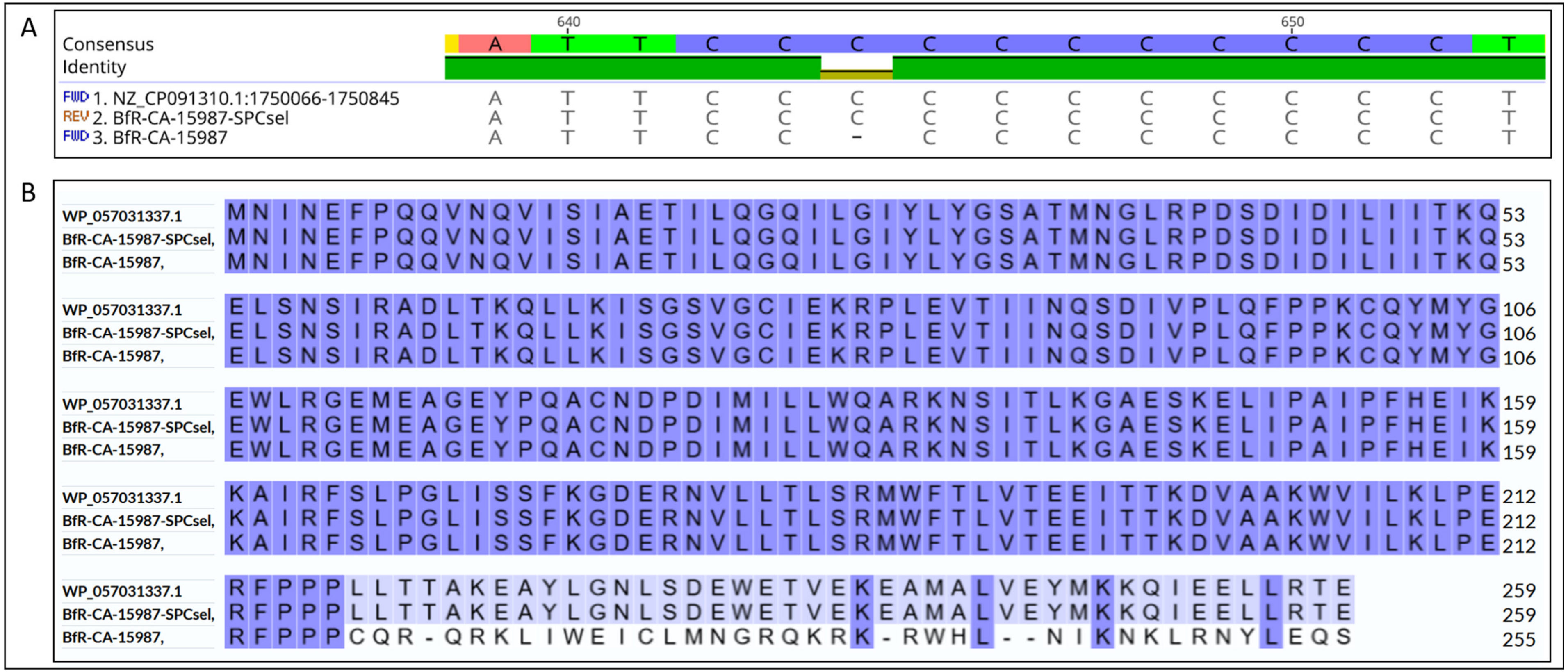

Figure S7. *aad9* is a phase-variable gene frequently inactivated by frame-shifting. **A,** Gene alignment of the poly-C tract in reference gene *aad9* (NZ_CP091310.1) with the corresponding region in BfR-CA-15987 and the spectinomycin re-selected BfR-CA-15987-SPCsel; **B,** Protein alignment of reference Aad9 (WP_057031337.1) with translated *aad9* gene assemblies obtained from BfR-CA-15987 before and after re-selection (BfR-CA-15987-SPCsel) on spectinomycin. Re-selection induced insertion of 1 cytosine into the poly-C tract (leading to 11 cytosines) (**A**), resulting in the restoration of full-length protein (**B**). Nucleotide and protein alignments were created with Geneious Prime software (**A**) and the Uniprot Align tool (56) (**B**), respectively.

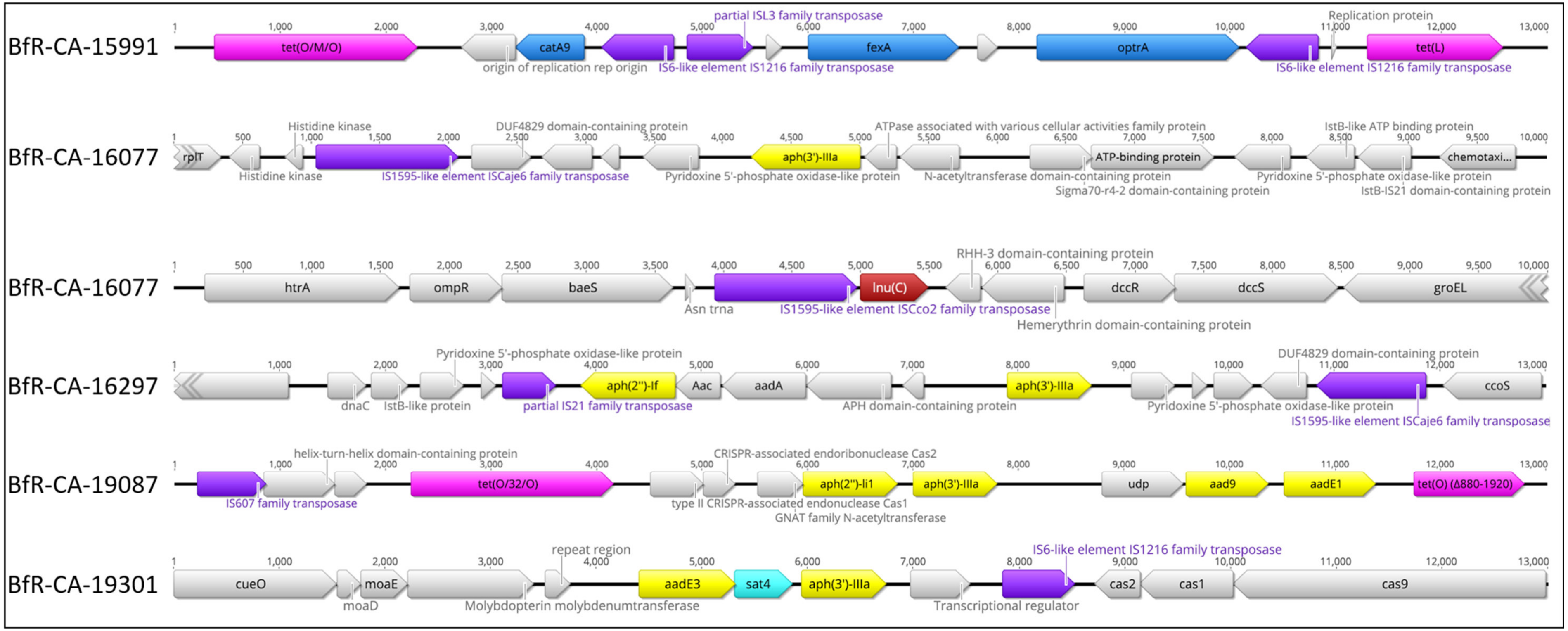

Figure S8. AMR genes in proximity to transposase genes not depicted in Figure 5 but part of Table 4. Transposase genes are marked in purple. AMR genes from different antimicrobial classes are depicted in different colors; blue, phenicol resistance genes (*catA, fexA, optrA*), red, *lnu*(C) genes; light purple, *tet* genes; yellow, aminoglycoside resistance genes; Grey arrows, non-AMR related genes.
